# Supplementary figures and images for: Do-It-Yourself digital archaeology: Introduction and practical applications of photography and photogrammetry for the 2D and 3D representation of small objects and artefacts
Source: PLoS One. 2022 Apr 15;17(4):e0267168. doi: 10.1371/journal.pone.0267168 (PMC9012351; doi:10.1371/journal.pone.0267168)

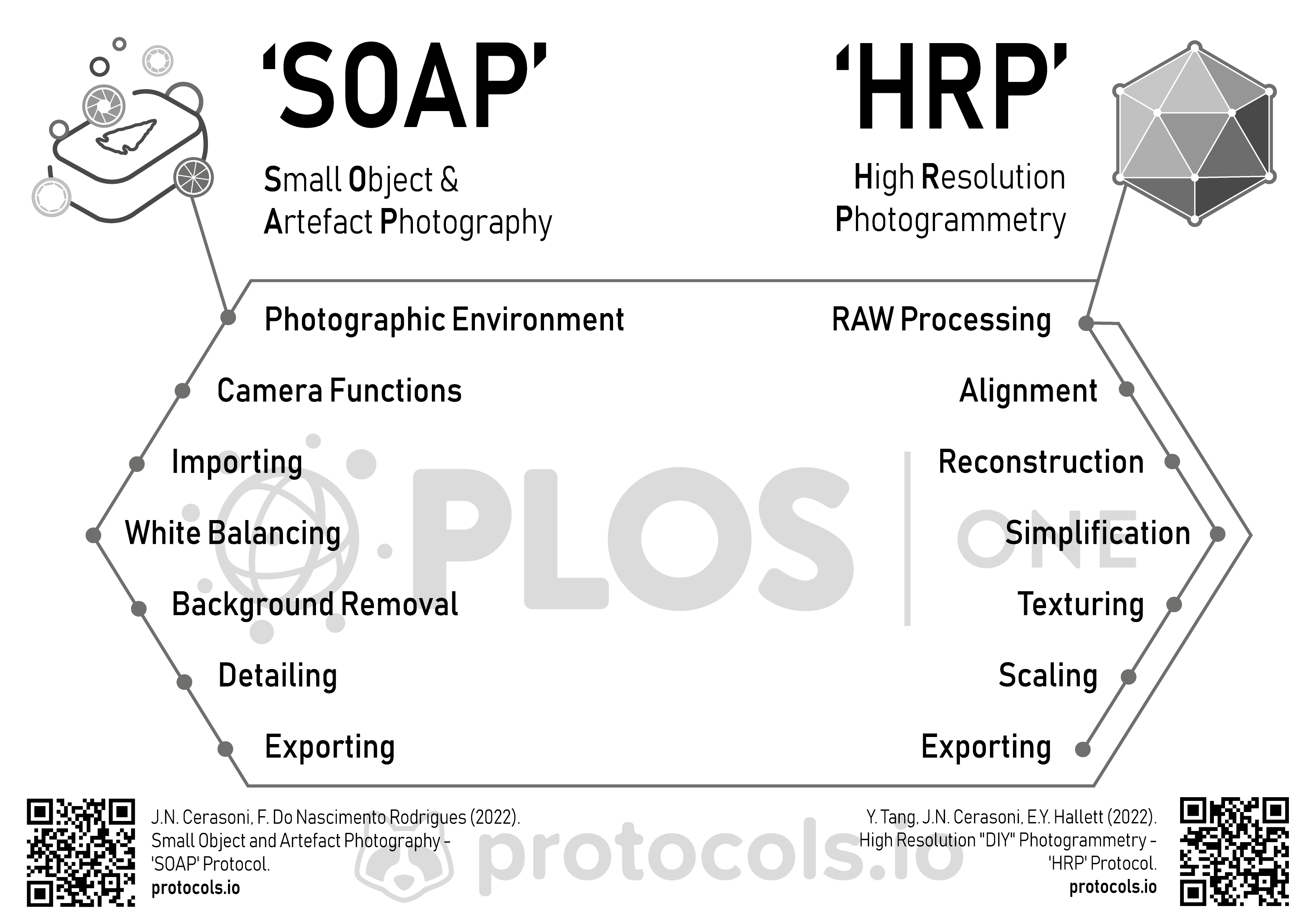

Supplement: S1 Fig — (PNG) [file pone.0267168.s001.png]
