# Supplementary material for: Do-It-Yourself digital archaeology: Introduction and practical applications of photography and photogrammetry for the 2D and 3D representation of small objects and artefacts
Source: PLoS One. 2022 Apr 15;17(4):e0267168. doi: 10.1371/journal.pone.0267168 (PMC9012351; doi:10.1371/journal.pone.0267168)
Supplement: S1 File — also available on protocols.io. (PDF) [file pone.0267168.s002.pdf]

Small Object and Artefact Photography - 'SOAP' Protocol V.3

Jacopo Niccolo Cerasoni[1], Felipe Do Nascimento Rodrigues[2]

[1]Pan African Evolution Research Group, Max Planck Institute for the Science of Human History, Jena, Germany;

[2]Centre for the Archaeology of the Americas, Department of Archaeology, University of Exeter

3

Mar 08, 2022

1

dx.doi.org/10.17504/protocols.io.b53zq8p6

Jacopo Niccolo Cerasoni

Photography is among one of the most widely used methods in scientific publications to efficiently and objectively communicate morphological, technological and aesthetic characteristics of any object. Particularly, in the fields of archaeology and anthropology, the study of small objects and artefacts is fundamental for the better understanding of past and present human activities. For these purposes, photography offers a method for researchers and alike to use photographs as objective evidence for their findings, results and interpretations. Here, the Small Object and Artefact Photography (SOAP) protocol is presented. This protocol aims to offer a standardised and full-encompassing method for the most objective and scientifically reproducible application of photography for academic purposes. The protocol follows an optimised step-by-step method, presenting the practice of small object and artefact photography from the initial set up of the equipment, the best methods for camera handling and functionality use, to the application of post-processing softwares. This method has been developed for anyone interested in producing clear and high quality photographs for a multitude of applications, from academic and scientific publications to public outreach.

DOI

dx.doi.org/10.17504/protocols.io.b53zq8p6

Jacopo Niccolo Cerasoni, Felipe Do Nascimento Rodrigues 2022. Small Object and Artefact Photography - 'SOAP' Protocol. **protocols.io**
https://dx.doi.org/10.17504/protocols.io.b53zq8p6
Jacopo Niccolo Cerasoni

archaeology, photography, methodology, graphic representation, artefact, anthropology

protocol ,

Citation: Jacopo Niccolo Cerasoni, Felipe Do Nascimento Rodrigues Small Object and Artefact Photography - 'SOAP' Protocol
https://dx.doi.org/10.17504/protocols.io.b53zq8p6

This is an open access protocol distributed under the terms of the **Creative Commons Attribution License** (https://creativecommons.org/licenses/by/4.0/), which permits unrestricted use, distribution, and reproduction in any medium,

Mar 08, 2022

Mar 08, 2022

59225

In the following protocol, typographical emphases and brackets have been used for software and in-text references.
They are: (1) software features and tools written in bold, e.g. **lock** or **Rectangle Tool**; (2) "layers" in "quotation marks", e.g. "background"; (3) references to steps of the protocol in *italics*, e.g. *step 21*; (4) <keyboard buttons> in <angle markers>, e.g. <right click> or <Del>; and (5) computational workflows linked by angle markers (>), e.g. <right click> > Arrange > Bring to Back.

Please note: This protocol has been developed for Adobe® Photoshop®, being specifically developed using Adobe® Phtshop® 2021. Nevertheless, previous Adobe® Photoshop® versions and other vector graphics softwares can be used (e.g. GIMP®). If previous versions or different softwares are being used, some functionalities will have to be accessed and applied differently.

List of materials for the execution (*Steps 1-13*) of this protocol:

1x Canon EOS 4000D Camera
1x Canon Zoom Lens EF-S 18-55 mm 1: 3.5 - 5.6
1x Manfrotto 035 Ftc Super Clamp
1x Manfrotto 037 Camera Mounting Stud
1x Manfrotto Single Arm 2 Section with Camera Bracket
1x Konig 40x40 cm Foldable Mini Photo Studio Kit (2x Light stand, 2x 800lm Lamp, 4x Backdrop, Carrying bag)
1x Plastic Black and White Photographic Scale

Collect Equipment

1 Collect and prepare equipment (see materials section for full list of equipment).

Citation: Jacopo Niccolo Cerasoni, Felipe Do Nascimento Rodrigues Small Object and Artefact Photography - 'SOAP' Protocol https://dx.doi.org/10.17504/protocols.io.b53zq8p6

This is an open access protocol distributed under the terms of the **Creative Commons Attribution License** (https://creativecommons.org/licenses/by/4.0/), which permits unrestricted use, distribution, and reproduction in any medium,

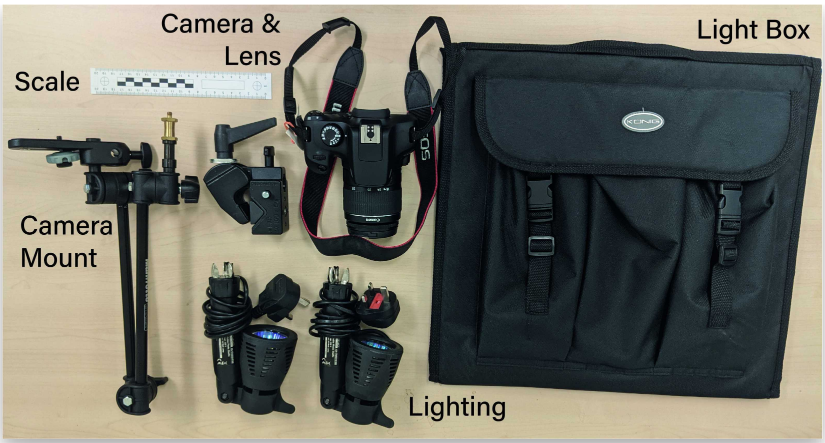

Basic equipment necessary for photographing small objects and artefacts.

1.1 Arguably the most important part of a camera is the lens. Before continuing with this method, we suggest the reader understand what type of lenses exist and which one works best for its use. In this case, we suggest using a macro lens. Find below definitions and descriptions of Macro Lens and commonly used standard zoom lens:

Macro Lens: A Macro Lens is a type of lens that allows very close up shots of the subject. In many cases, it requires close proximity to the subject being photographed but in return, it offers high levels of detail, which are difficult to be acquired with regular lens. This in turn, makes macro lens ideally suited for small object and artefacts photography.

Standard Zoom Lens: Standard Zoom Lens is a highly variable type of lens, allowing for the capture of landscapes, people, buildings and small objects and artefacts. Although it may not perform as well as a macro lens, its flexibility makes it an ideal lens for many archaeology professionals, projects and institutions, hence, one of the most commonly available lens types.

Ultimately, the choice of a lens should be reflected against the questions and needs of the archaeologist/photographer. If highly detailed close-up photographs are required, a Macro Lens is recommended, however, if illustrative photographs are required, a Standard Zoom Lens is more than capable to achieve good and usable photographs.

Setting Photographic Environment

2 Select lens (ideally macro lens), lock camera on a tripod, camera mount or copy stand and place on light box (if available). Ideally use a macro lens.

Please note that the camera support greatly depends on the surroundings where the photographic station has been set up. Tripods work best on open spaces. On tighter spaces,

Citation: Jacopo Niccolo Cerasoni, Felipe Do Nascimento Rodrigues Small Object and Artefact Photography - 'SOAP' Protocol https://dx.doi.org/10.17504/protocols.io.b53zq8p6

This is an open access protocol distributed under the terms of the **Creative Commons Attribution License** (https://creativecommons.org/licenses/by/4.0/), which permits unrestricted use, distribution, and reproduction in any medium,

photography arms with a clamp to attach to stable surfaces (such as tables) are more suitable.

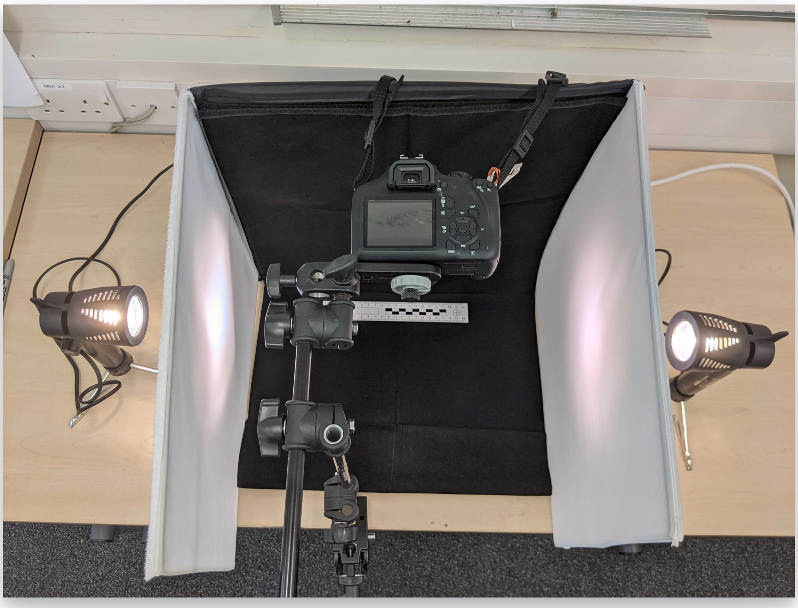

Camera locked into photography arm. Make sure all connecting sections are tight for a stable photograph.

2.1 To ease the use of the camera, a remote control can be used (if the camera does not include one, remote control can be accessed via WiFi or Bluetooth; both Android and iOS universal applications exist for this purpose).

Citation: Jacopo Niccolo Cerasoni, Felipe Do Nascimento Rodrigues Small Object and Artefact Photography - 'SOAP' Protocol https://dx.doi.org/10.17504/protocols.io.b53zq8p6

This is an open access protocol distributed under the terms of the **Creative Commons Attribution License** (https://creativecommons.org/licenses/by/4.0/), which permits unrestricted use, distribution, and reproduction in any medium,

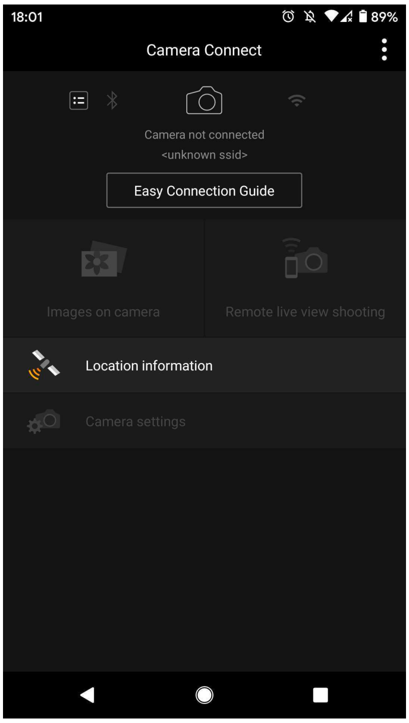

Canon Camera Connect iOS software used to capture the images for this protocol (*steps 13*).

> In case remote control is not available, the use of the camera timer (2-10s) is recommended as an option to avoid blurriness from shaking when operating it on the tripod/mount.

2.2 If a tripod or camera mount cannot be used, make sure to grip the camera properly, following these instructions:

- Use both hands (right hand on camera grip, left hand under lens)
- Stabilise whole body (rest camera-holding arm on knee or solid surface, or lean whole body against surface)

3 Place background material. Satin paper or velvet are preferred given their non-reflective properties.

Citation: Jacopo Niccolo Cerasoni, Felipe Do Nascimento Rodrigues Small Object and Artefact Photography - 'SOAP' Protocol https://dx.doi.org/10.17504/protocols.io.b53zq8p6

This is an open access protocol distributed under the terms of the **Creative Commons Attribution License** (https://creativecommons.org/licenses/by/4.0/), which permits unrestricted use, distribution, and reproduction in any medium,

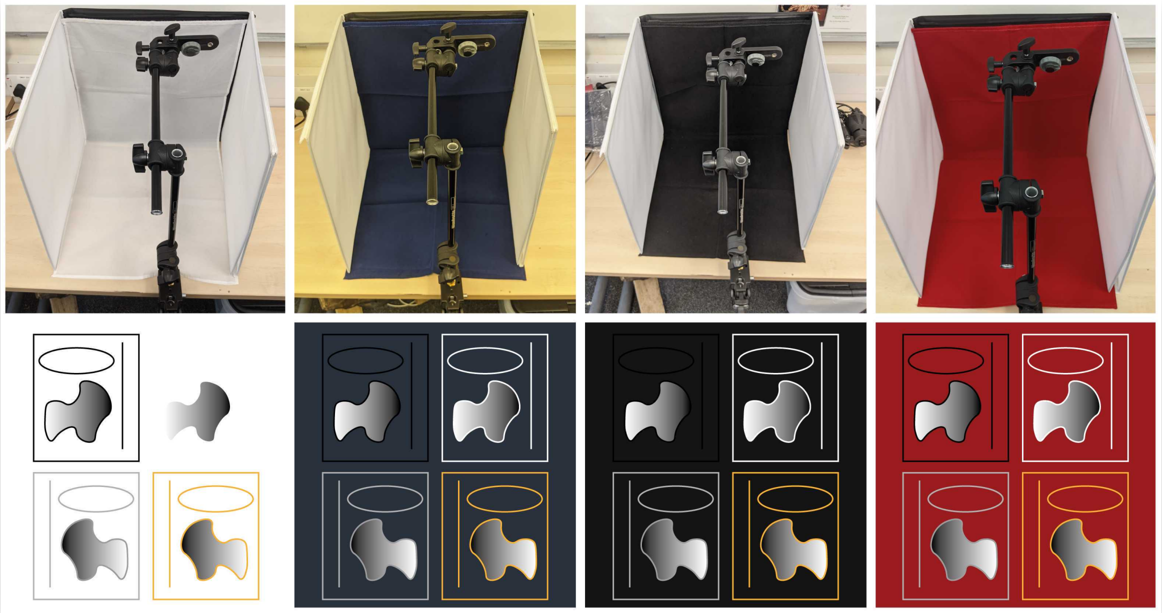

Examples of different coloured backgrounds and a schematic representation of interaction between background colours and differently coloured objects.

3.1 The colour of the background should be complementary to the main colour of the artefact or object being photographed. The best colour for the background will make the subject stand out. This will make it easier later in the process to colour balance the image and remove the background digitally.

4 Place artefact flat onto the workspace.

4.1 If the artefact does not sit flat due to its irregular shape, use an appropriate amount of modelling clay wrapped in plastic wrap so to create a modellable support surface.

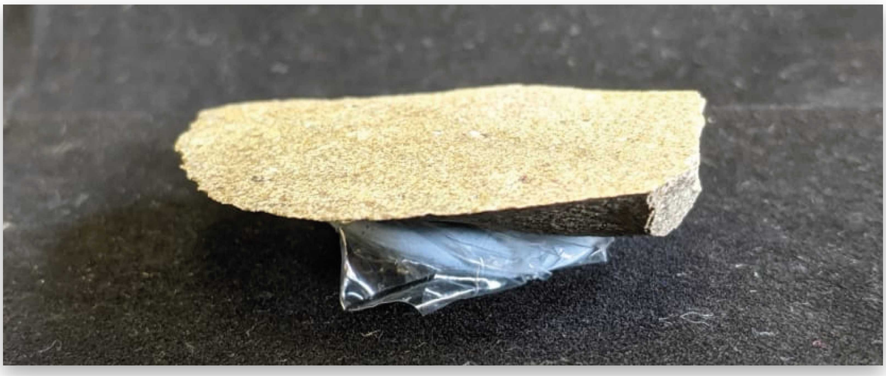

Example of lithic flake positioned on modelling clay wrapped in plastic wrap.

Citation: Jacopo Niccolo Cerasoni, Felipe Do Nascimento Rodrigues Small Object and Artefact Photography - 'SOAP' Protocol https://dx.doi.org/10.17504/protocols.io.b53zq8p6

This is an open access protocol distributed under the terms of the **Creative Commons Attribution License** (https://creativecommons.org/licenses/by/4.0/), which permits unrestricted use, distribution, and reproduction in any medium,

5 With lamps or other light-sources set up direct and diffused lighting from one or more sides and the top. Try to prevent major shadowing on the background surface.

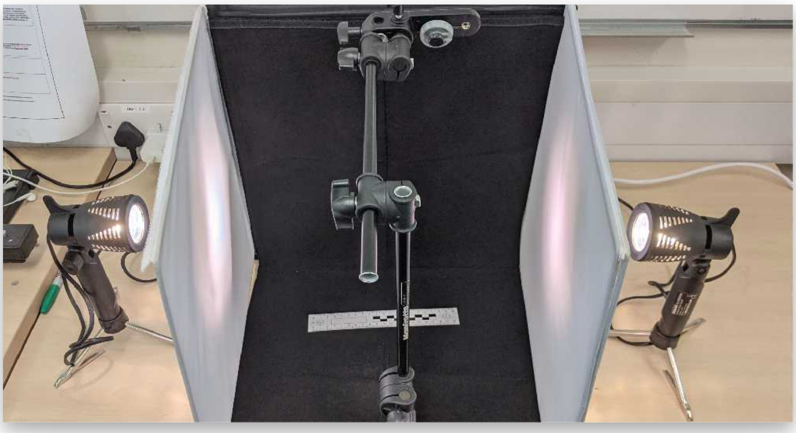

Example of lateral diffused lighting.

Setting Camera Functions

6 Select image raw format.

RAW image formats (CR2, CRW, NEF, PEF...) are always preferred as they undergo the least amount of digital processing, with little to no loss of visual details.

Other formats can also be used if very high resolution photos are not necessary, and they are:

1. Tagged Image Files (TIF/TIFF): Large files which do not loose much visual quality by using the so-called "loss-less" compression.
2. JPG/JPEG: Standard and default format for many application. Considerable compression, resulting in lower quality and smaller files.

In photography, **compression** is the process of minimizing the digital size of a graphical element (photograph) without degrading the quality of an image to an unacceptable level. Due to this, both RAW and TIF formats are very good for archival purposes.

Citation: Jacopo Niccolo Cerasoni, Felipe Do Nascimento Rodrigues Small Object and Artefact Photography - 'SOAP' Protocol https://dx.doi.org/10.17504/protocols.io.b53zq8p6

This is an open access protocol distributed under the terms of the **Creative Commons Attribution License** (https://creativecommons.org/licenses/by/4.0/), which permits unrestricted use, distribution, and reproduction in any medium,

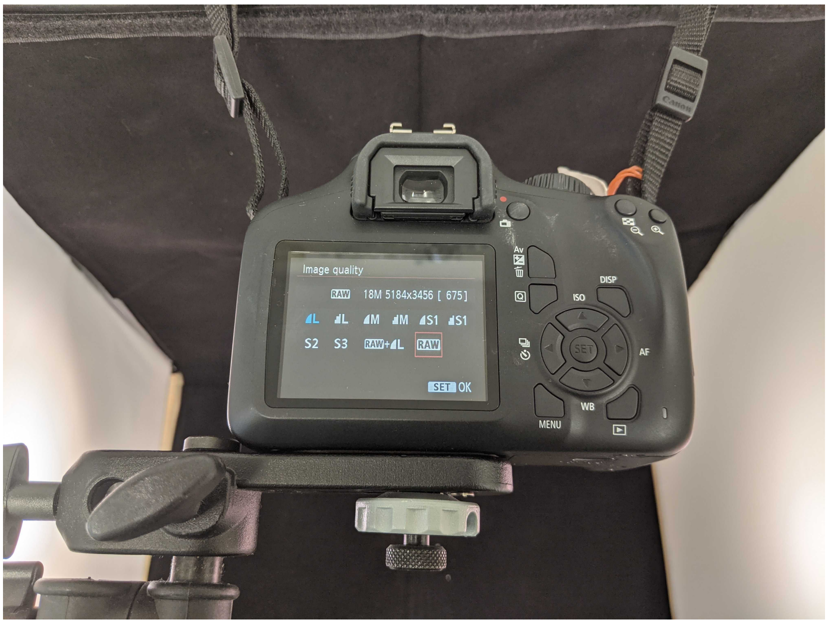

Image quality format options available on the camera model used (Canon EOS 4000D). Please note that the location will vary based on camera model and manufacturer.

7 Select photographic mode.

Generally, manual mode is preferred as it gives the highest degree of freedom for adjusting the various functions and settings.

In some cases, Aperture or Time modes can also be used as they offer the possibility to adjust just one of the two respecting settings. In such cases, all the other camera functions and settings (i.e. ISO, exposure, focus...) will be set automatically.

Automatic mode should never be used when photographic small objects and artefacts, as each photograph taken could result in different values assigned for each setting, therefore resulting in technically inconsistent photographs.

Citation: Jacopo Niccolo Cerasoni, Felipe Do Nascimento Rodrigues Small Object and Artefact Photography - 'SOAP' Protocol https://dx.doi.org/10.17504/protocols.io.b53zq8p6

This is an open access protocol distributed under the terms of the **Creative Commons Attribution License** (https://creativecommons.org/licenses/by/4.0/), which permits unrestricted use, distribution, and reproduction in any medium,

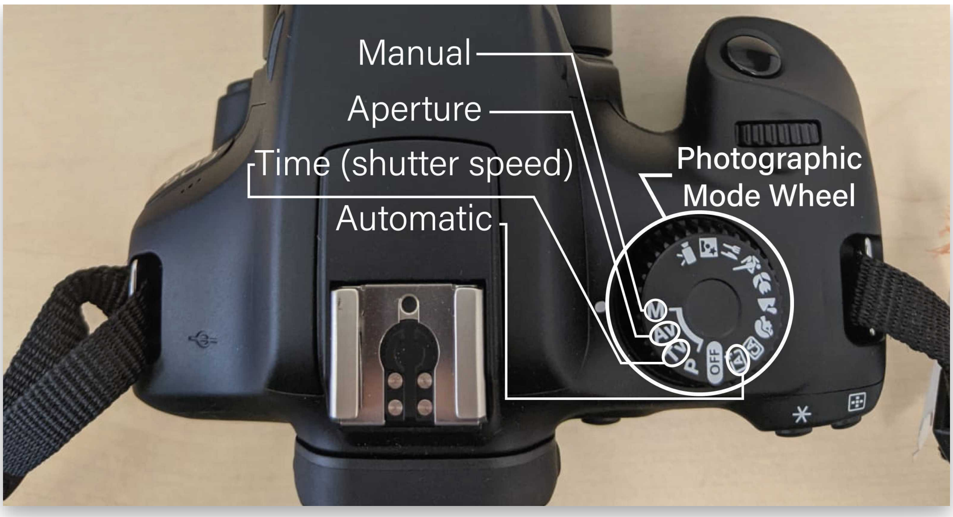

Main photographic modes described in *Step 7*.

8 White Balance

White balance is the adjustment of the colour temperatures captured by the lenses. On the camera, this is a process that can be adjusted in three ways:

1. Automatic: Auto adjusted during capture

2. Pre-set: Predetermined categories built in the camera (e.g. 'Cloudy', 'Incandescent', 'Direct Sunlight')

3. Manual: Some cameras come with the option to manually choose a colour temperature (Values in Kelvin)

These on-camera options can be useful if a quicker acquisition and processing is required.

Lastly, White Balance can be also changed on the Post Processing phase by: manually adjusting the values on post processing software (see step 18).

9 Adjust Exposure.

Exposure is the amount of light which reaches the camera sensor. This is dependent on the relationship between the three main functions discussed previously, which include:

1. Aperture
2. Shutter Speed (or Time Value)
3. ISO

Adjusting these three separate functions will result in a certain amount of light reaching the sensor. Furthermore, exposure can be further altered using the in-camera exposure function.

Citation: Jacopo Niccolo Cerasoni, Felipe Do Nascimento Rodrigues Small Object and Artefact Photography - 'SOAP' Protocol https://dx.doi.org/10.17504/protocols.io.b53zq8p6

This is an open access protocol distributed under the terms of the **Creative Commons Attribution License** (https://creativecommons.org/licenses/by/4.0/), which permits unrestricted use, distribution, and reproduction in any medium,

Although it is possible to set a determined exposure value either as positive (lighter) or negative (darker) values, the **exposure setting should always be set at 0** when possible. This is because further digital processing will be applied when exposure is directly set onto the camera, decreasing image quality. Exposure should therefore be adjusted only by **regulating aperture**, **shutter speed** and **ISO**.

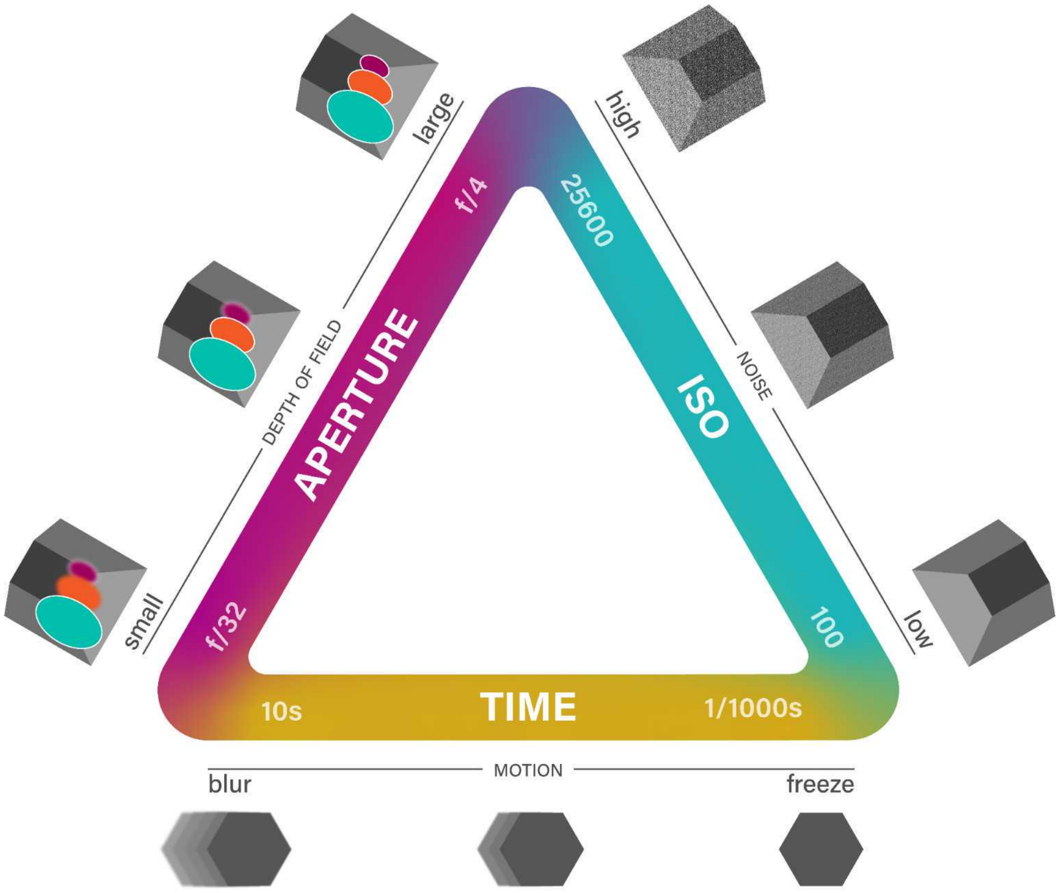

'Exposure Triangle' - Graphical representation of the relationship between the three main variables influencing image capture and described in *Step 9.* Time can also be called Shutter Speed (*Step 12*).

10 Set the depth of field.

The depth of field will determine what part of the subjects in the frame will be in focus. This will be dependent by the interaction of two settings:

1. Focal length
2. Aperture

By adjusting the focal length and the aperture it is possible to control which portion of the subject will be in focus. This can therefore result in a single flat plane being in focus, or a full

Citation: Jacopo Niccolo Cerasoni, Felipe Do Nascimento Rodrigues Small Object and Artefact Photography - 'SOAP' Protocol https://dx.doi.org/10.17504/protocols.io.b53zq8p6

This is an open access protocol distributed under the terms of the **Creative Commons Attribution License** (https://creativecommons.org/licenses/by/4.0/), which permits unrestricted use, distribution, and reproduction in any medium,

volumetric object being fully in focus.

10.1 Focal length is the measure of distance between the point of convergence of the lens and the sensor.

It is measured in millimetres (mm), and it defines how much of the scene will be captured. A small focal length will result in a wider angle of view, and vice versa.

The focal length is determined by the **focal variation of the lens** used. Generally, **macro lenses** are preferred, as they have a focal length between 30-55mm. This specific focal length range creates **crisp** and **sharp images** of small objects at **extremely close range**.

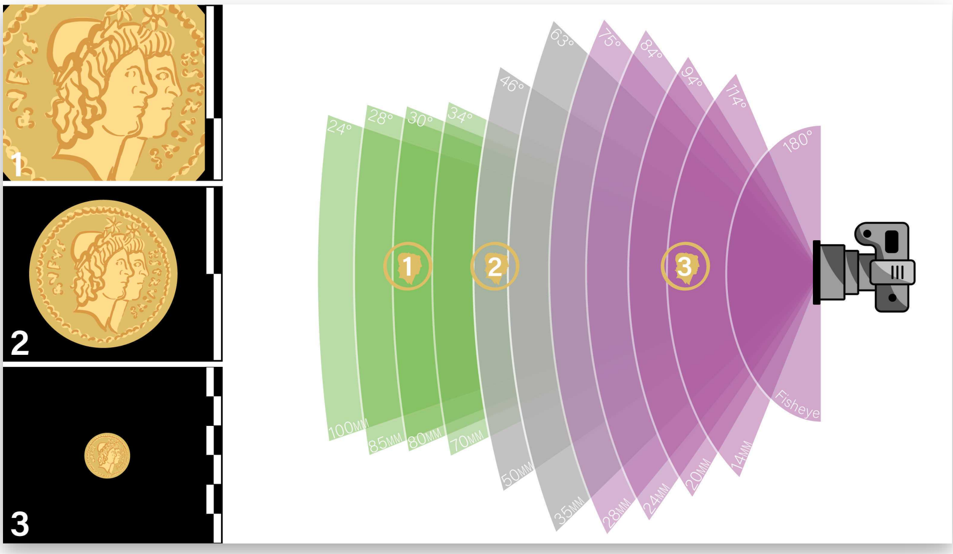

On the right, a schematic representation of the various focal length with respective angle of view and focal length measurement. On the left, an example of three views of the same artefact using three different focal lengths.

10.2 Aperture controls the opening that lets light in the lens.

It is expressed in f-stops (larger the number, smaller the opening)

Aperture affects depth of field as follows:

- Larger opening -> shallower depth of field
- Smaller opening -> larger depth of field

Citation: Jacopo Niccolo Cerasoni, Felipe Do Nascimento Rodrigues Small Object and Artefact Photography - 'SOAP' Protocol https://dx.doi.org/10.17504/protocols.io.b53zq8p6

This is an open access protocol distributed under the terms of the **Creative Commons Attribution License** (https://creativecommons.org/licenses/by/4.0/), which permits unrestricted use, distribution, and reproduction in any medium,

For small object and artefact photography, **aperture** will be **dependent on the volumetric profile** of the subject. Generally a large aperture can be used, however, if a deeper or more volumetric object has to be photographed, a smaller opening (therefore higher f-stop value) will be required.

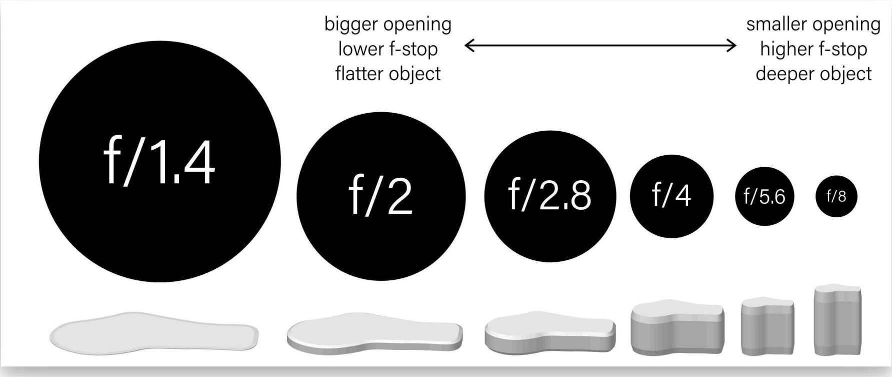

Diagram of the relationship between aperture and volumetric profile of the subject.

11 Select ISO (International Organisation for Standardization)

This is the light sensitivity of the sensor. A higher ISO means the lens will be more light sensitive, therefore increasing the image noise.

ISO is expressed in arbitrary doubling numbers, from 50 to 25600 (50, 100, 200, 400, etc.).

Ideally, the **lowest possible ISO** should be used so to keep **image noise at a minimum**. ISO values larger than 200 should never be used due to the considerable sensitivity of the sensor and therefore loss of quality.

12 Set the shutter speed.

The shutter speed, or time value, is the length of time the camera shutter is open. It is therefore responsible for:

1. the brightness level of the photograph
2. the degree of blurring motion or freezing action in the case the object or camera are not still.

It is expressed in fractions of a second or full seconds.

Citation: Jacopo Niccolo Cerasoni, Felipe Do Nascimento Rodrigues Small Object and Artefact Photography - 'SOAP' Protocol https://dx.doi.org/10.17504/protocols.io.b53zq8p6

This is an open access protocol distributed under the terms of the **Creative Commons Attribution License** (https://creativecommons.org/licenses/by/4.0/), which permits unrestricted use, distribution, and reproduction in any medium,

For small object and artefact photography, the blurring motion should be absent, therefore the **shutter speed** will have to be **adjusted depending on the environmental lighting** (i.e. intensity and diffusion of lighting), so to photograph the subject at a reasonable lighting level.

13 Set the focus.

Focus is the adjustment of the lens to find the maximum resolution, sharpness and contrast for the chosen subject.

Focus can be achieved either with a Manual or Automatic Focus mode. When photographing still macro subjects, such as in this case, Manual Focus is preferred as it offers full control to select and focus upon the surfaces of interest.

Manual focus can be adjusted by selecting the **Manual Focus function** (either directly onto lens or through camera settings), and adjusting the **Focus Ring** (outer lens portion) and **Zoom ring** (inner lens portion).

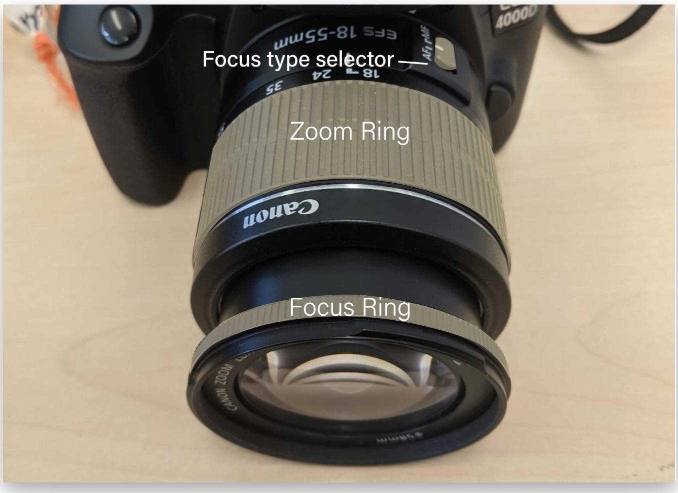

Example of location of focus type selector, zoom ring, and focus ring.

Note that some lenses do not have both focus and zoom ring. In these cases, focus is adjusted by moving the camera closer/further to the object. The example illustrated above applies to most, but not all lenses.

Citation: Jacopo Niccolo Cerasoni, Felipe Do Nascimento Rodrigues Small Object and Artefact Photography - 'SOAP' Protocol https://dx.doi.org/10.17504/protocols.io.b53zq8p6

This is an open access protocol distributed under the terms of the **Creative Commons Attribution License** (https://creativecommons.org/licenses/by/4.0/), which permits unrestricted use, distribution, and reproduction in any medium,

14 Experiment with different settings.

Rarely the perfect settings are found on the first try, and environmental variables can constantly change. For this reason, take several photos with different values for each setting.

> Note that the photographs shown on the camera screen are not always reliable. It is therefore advised to double check the photographs on a computer regularly.

Below are a series of photographs taken of the same artefact, keeping the environmental variables and photographic equipment consistent.

14.1

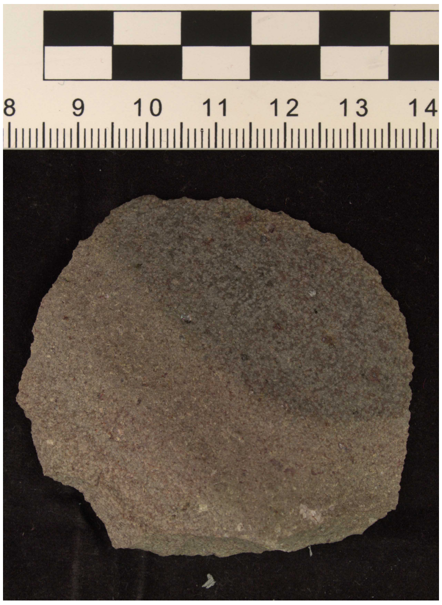

IMG_0914

Focal Length - 47mm

Aperture (F/stop) - f/8

Exposure time (shutter speed) - 1/3 sec.

ISO - ISO-100

Citation: Jacopo Niccolo Cerasoni, Felipe Do Nascimento Rodrigues Small Object and Artefact Photography - 'SOAP' Protocol https://dx.doi.org/10.17504/protocols.io.b53zq8p6

This is an open access protocol distributed under the terms of the **Creative Commons Attribution License** (https://creativecommons.org/licenses/by/4.0/), which permits unrestricted use, distribution, and reproduction in any medium,

14.2

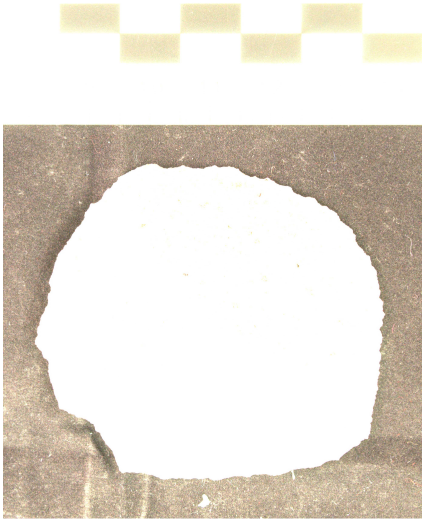

IMG_0915

Focal Length - 47mm

Aperture (F/stop) - f/8

Exposure time (shutter speed) - 1/3 sec.

ISO - 6400

14.3

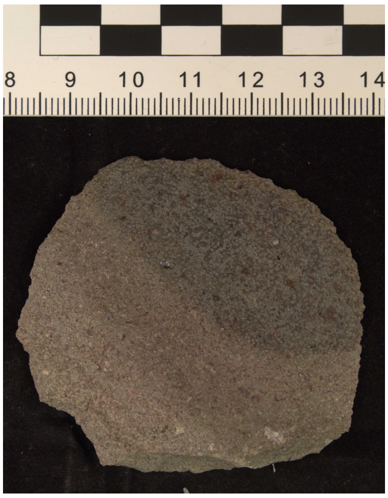

IMG_0916

Focal Length - 50mm

Aperture (F/stop) - f/8

Exposure time (shutter speed) - 1/4 sec.

Citation: Jacopo Niccolo Cerasoni, Felipe Do Nascimento Rodrigues Small Object and Artefact Photography - 'SOAP' Protocol
https://dx.doi.org/10.17504/protocols.io.b53zq8p6

This is an open access protocol distributed under the terms of the **Creative Commons Attribution License**
(https://creativecommons.org/licenses/by/4.0/), which permits unrestricted use, distribution, and reproduction in any medium,

ISO - 200

Importing

15 Open Adobe® Photoshop®.

16 Select: **Open** > choose photograph of choice.

16.1 If the original image was in RAW format (depending on brand of camera used, RAW images will have different names and encodings, e.g. DCR, CRW, CR2, CR3, NEG, ARW), the software Adobe® Camera Raw® will automatically open.

do not modify the photograph on Adobe® Camera Raw®, click **open** to import the photograph on Adobe® Photoshop®.

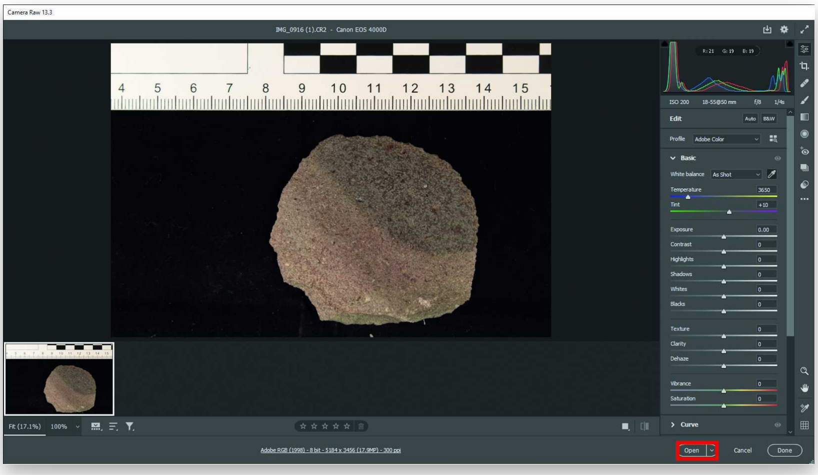

Snapshot of Adobe® Camera Raw® showing location of **open** in red.

17 The photograph will automatically be imported as the "background" layer.

Unlock this layer by selecting the **lock** symbol. The layer will then be named "layer 0" and fully workable when selected.

Citation: Jacopo Niccolo Cerasoni, Felipe Do Nascimento Rodrigues Small Object and Artefact Photography - 'SOAP' Protocol https://dx.doi.org/10.17504/protocols.io.b53zq8p6

This is an open access protocol distributed under the terms of the **Creative Commons Attribution License** (https://creativecommons.org/licenses/by/4.0/), which permits unrestricted use, distribution, and reproduction in any medium,

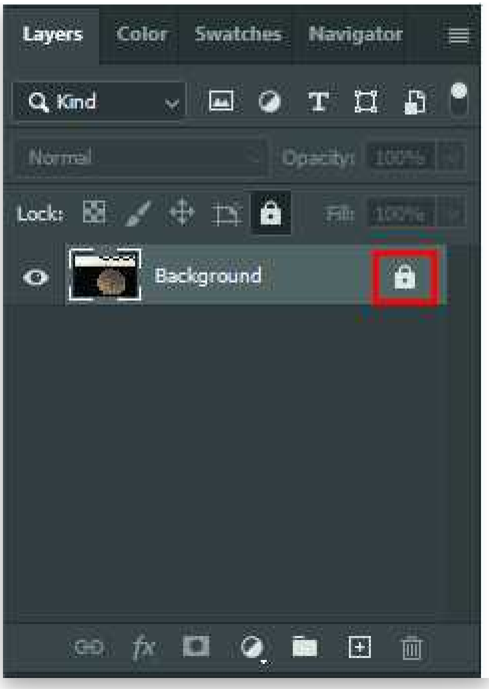

Snapshot of the **layers** tab, with the **lock** symbol marked in red.

17.1 Note: To make a layer selected, click on the layer of choice on the **layers** tab. This will make the layer on the **layers** tab light grey in colour, and any action or selection will apply to the specific layer.

White Balancing

18 White balancing is the process of adjsting colour casts of the photograph. This will therefore adjust all color curves based on what is manually selected as a pure white, realistically adjusting the photograph's colours to their true tone and hue.

Once the selected photograph is imported, the first step is to balance the whites, greys and blacks.

> Please note that the following method describes a non-linear colour adjustment of pixels within a photograph. Studies have shown that this method can at times be non-ideal for scientific purposes (Rossner and Yamada 2004; https://doi.org/10.1083/jcb.200406019). Alternatively, photo filters can be used to achieve a linear adjustment, although they require subjective judgement.
>
> If the method described here is used for scientific publication, we suggest disclosing that this manipulation technique was used.

Citation: Jacopo Niccolo Cerasoni, Felipe Do Nascimento Rodrigues Small Object and Artefact Photography - 'SOAP' Protocol https://dx.doi.org/10.17504/protocols.io.b53zq8p6

This is an open access protocol distributed under the terms of the **Creative Commons Attribution License** (https://creativecommons.org/licenses/by/4.0/), which permits unrestricted use, distribution, and reproduction in any medium,

19 Select: **Image** > **Adjustments** > **Curves...**

19.1 Use the **White**, **Grey** and **Black Balancing Tools** to adjust the white balance of the photograph.

This will be done by selecting each tool and clicking on an area of the photograph with its respective tone.

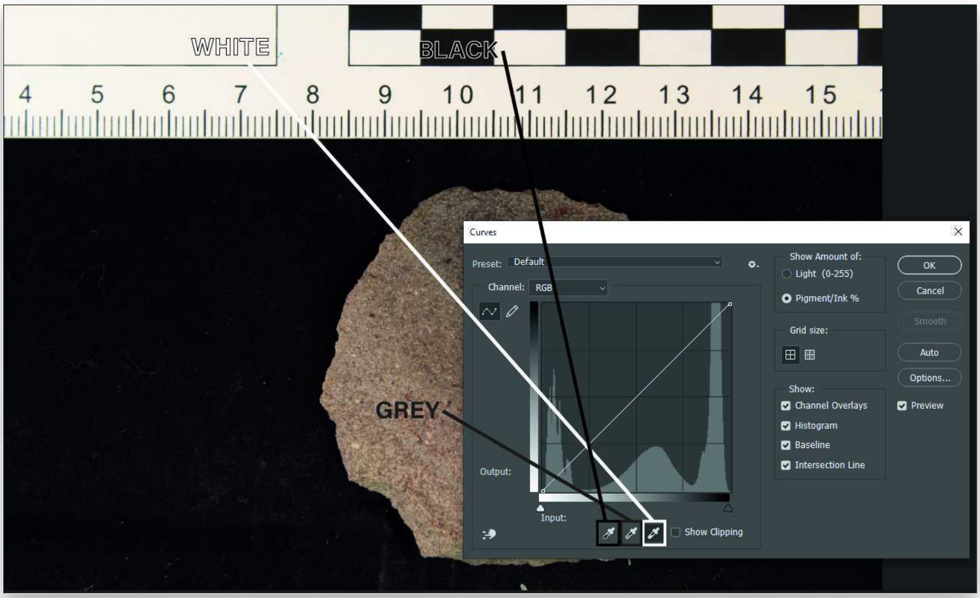

Snapshot of location of **white**, **grey** and **black balancing tools** and areas of respective colour tones.

19.2 Select **OK** to confirm white balancing.

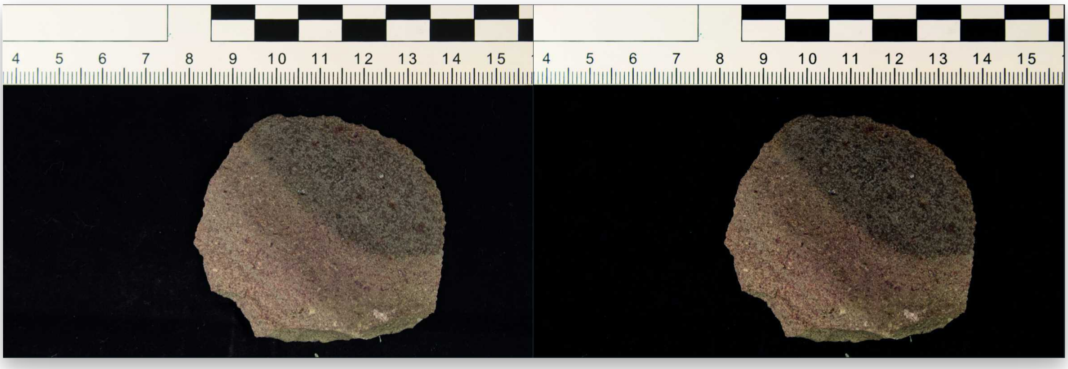

Result of white balancing. On the left photograph before steps *17* and *18*, on the right final product.

Citation: Jacopo Niccolo Cerasoni, Felipe Do Nascimento Rodrigues Small Object and Artefact Photography - 'SOAP' Protocol https://dx.doi.org/10.17504/protocols.io.b53zq8p6

This is an open access protocol distributed under the terms of the **Creative Commons Attribution License** (https://creativecommons.org/licenses/by/4.0/), which permits unrestricted use, distribution, and reproduction in any medium,

20 For automatic background removal: **Select** > **Subject**

20.1 Most times the subject will be appropriately selected and isolated. If, however, the selection area is incorrect (with extra or missing portions), the **Lasso tool** can be used to add or remove areas.

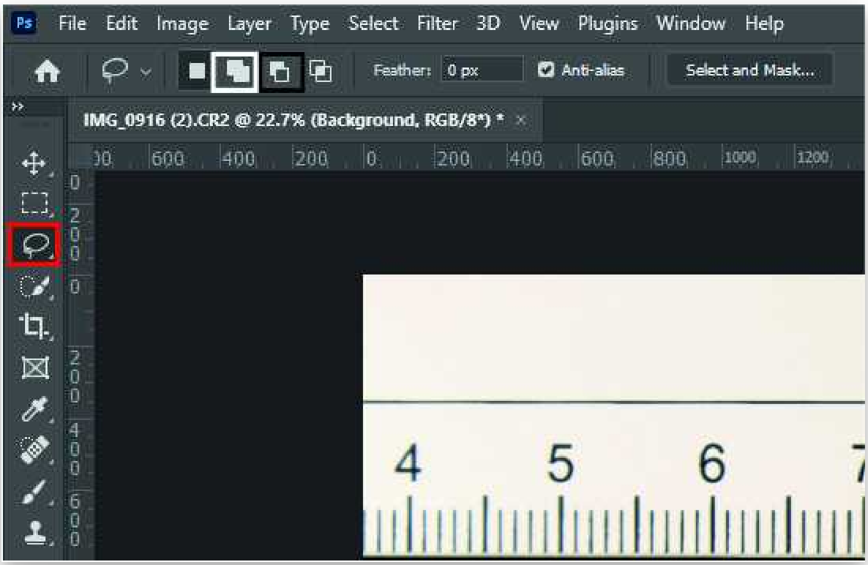

Location of **Lasso tool**, with respective **addition** (in white) icon to add missing selected areas, and **subtraction** (in black) icon to remove extra selected areas.

20.2 Double check that edges of selection areas are consistent with the original edges of the object or artefact.

21 Once the object has been selected properly, you can enhance the edges so to have a cleaner looking product. This is done with the **feathering** function, as it will create a transparency gradient and create a less "abrupt" edge.

To do so, right click on the isolated subject and select **Feather...** > select a feathering radius of maximum 3 pixels. Then click **OK**.

Citation: Jacopo Niccolo Cerasoni, Felipe Do Nascimento Rodrigues Small Object and Artefact Photography - 'SOAP' Protocol https://dx.doi.org/10.17504/protocols.io.b53zq8p6

This is an open access protocol distributed under the terms of the **Creative Commons Attribution License** (https://creativecommons.org/licenses/by/4.0/), which permits unrestricted use, distribution, and reproduction in any medium,

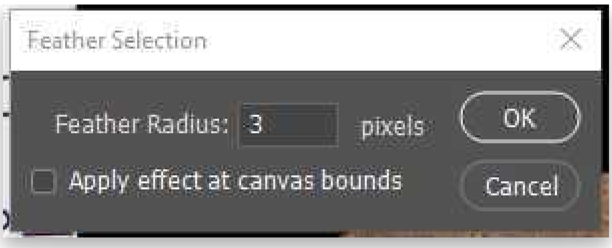

Snapshot of **Feather** function.

22 Finally, using the **Lasso Tool**, <right click> on the subject, and select **Layer via copy...**

This will create a separate layer, with the final subject correctly isolated and ready for finalisation.

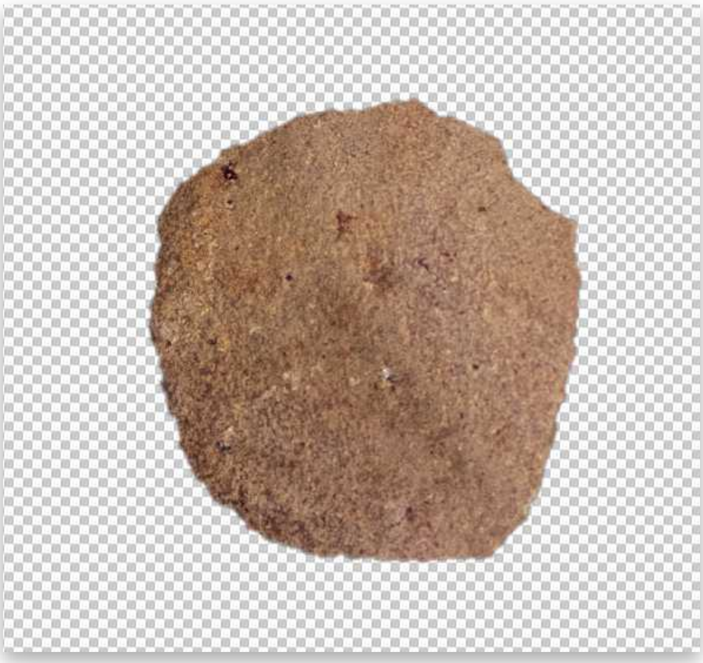

Result after background removal.

Detailing and Composition

23 To include a background, create a new "layer", and draw the background using the **Rectangle Tool**. Keeping in mind *Step 3*, select the best suited colour as a background.

24 To add a scale activate the "layer" of original photograph. Use the **Rectangle Tool** or **Line Tool** to trace the original photographed scale to the desired length, hence creating a new

Citation: Jacopo Niccolo Cerasoni, Felipe Do Nascimento Rodrigues Small Object and Artefact Photography - 'SOAP' Protocol https://dx.doi.org/10.17504/protocols.io.b53zq8p6

This is an open access protocol distributed under the terms of the **Creative Commons Attribution License** (https://creativecommons.org/licenses/by/4.0/), which permits unrestricted use, distribution, and reproduction in any medium,

object which will be the final scale of choice.

25 Using the **Rectangle Tool**, **Line Tool**, **Elipse Tool**, **Pen Tool**, or any other preferred tool, add any symbols of choice (e.g. dotted line for wear, arrow for percussion point)

26 Repeat *Steps 22-25* for any other subject of choice.

27 Once all the items of choice are included in a single canvas, position them in their final position and add connectors if you wish (using the **Line Tool** or **Rectangle Tool**).

> To help you with the positioning of the subjects in a single composition, you can use the Grid function. Also, the **Transform Tool** will let you resize objects. For the latter, make sure to resize while keeping the size ratio uniform (hold <Shift> key), and always resize the relevant scale together with the subject.

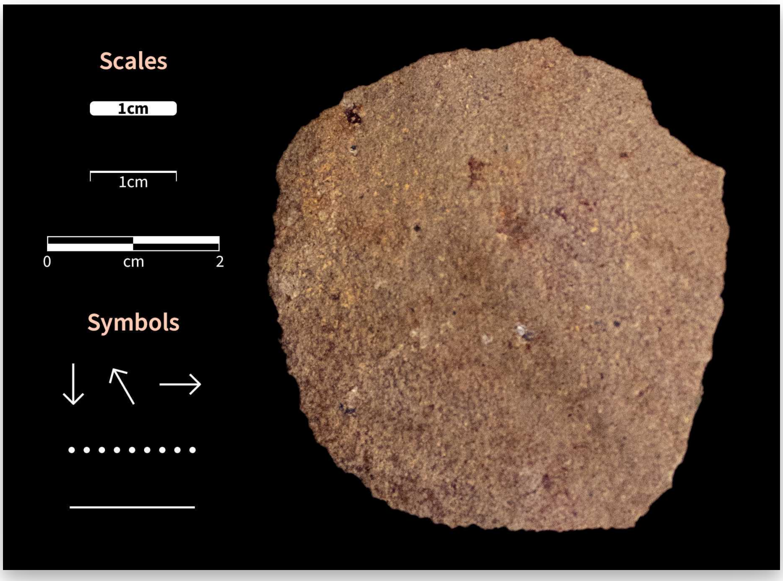

Example of scale formats and symbols.

Exporting

28 On Adobe® Photoshop® a variety of export formats can be selected. The most common are:

1. .TIF/TIFF (File > Save as... > Save as type: > TIFF)

Citation: Jacopo Niccolo Cerasoni, Felipe Do Nascimento Rodrigues Small Object and Artefact Photography - 'SOAP' Protocol https://dx.doi.org/10.17504/protocols.io.b53zq8p6

This is an open access protocol distributed under the terms of the **Creative Commons Attribution License** (https://creativecommons.org/licenses/by/4.0/), which permits unrestricted use, distribution, and reproduction in any medium,

2. .JPG (File > Export > Export as... > Save as type: > JPEG)

3. .PNG (File > Export > Export as... > Save as type: > PNG)

4. .PSD/.PDD/.PSDT (File > Save as... > Save as type: > Photoshop)

.TIFF, .JPG, .PNG are useful formats once the image is finished. However, it is recommended to have a version saved on the native format of the image processing software used (i.e. .PSD on photoshop, .XCF on GIMP or equivalent) as that allows further processing of the image if required.

Citation: Jacopo Niccolo Cerasoni, Felipe Do Nascimento Rodrigues Small Object and Artefact Photography - 'SOAP' Protocol https://dx.doi.org/10.17504/protocols.io.b53zq8p6

This is an open access protocol distributed under the terms of the **Creative Commons Attribution License** (https://creativecommons.org/licenses/by/4.0/), which permits unrestricted use, distribution, and reproduction in any medium,
