# Supplementary material for: Do-It-Yourself digital archaeology: Introduction and practical applications of photography and photogrammetry for the 2D and 3D representation of small objects and artefacts
Source: PLoS One. 2022 Apr 15;17(4):e0267168. doi: 10.1371/journal.pone.0267168 (PMC9012351; doi:10.1371/journal.pone.0267168)
Supplement: S2 File — also available on protocols.io. (PDF) [file pone.0267168.s003.pdf]

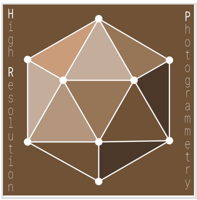

2

Mar 09, 2022

High Resolution "DIY" Photogrammetry - 'HRP' Protocol V.2

Yu Tang[1], Jacopo Niccolo Cerasoni[2,3], Emily Yuko Hallett[2]

[1]Cygames Inc., Shibuya-ku, Tokyo, Japan;
[2]Pan African Evolution Research Group, Max Planck Institute for the Science of Human History, Jena, Germany;
[3]Institute of Archaeological Sciences, Eberhard Karls University Tübingen, Tübingen, Germany

1

dx.doi.org/10.17504/protocols.io.b53xq8pn

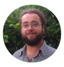

Jacopo Niccolo Cerasoni

Photogrammetry is a method of calculating the three-dimensional shape of an object from a set of images. The advantages of Photogrammetry include the ability to record the shape of an object in a short time and with high accuracy without contact. In addition, the generated model can be displayed without textures. Here, the High Resolution Photogrammetry method is presented, which describes the use of photogrammetric techniques to take pictures and generate models. This method aims to give a comprehensive and extensive description for the development of high resolution 3D models, merging the well known techniques used in academic and computer graphic fields, allowing anyone to independently produce high resolution and quantifiable models for any need.

DOI

dx.doi.org/10.17504/protocols.io.b53xq8pn

Yu Tang, Jacopo Niccolo Cerasoni, Emily Yuko Hallett 2022. High Resolution "DIY" Photogrammetry - 'HRP' Protocol. **protocols.io**
https://dx.doi.org/10.17504/protocols.io.b53xq8pn
Jacopo Niccolo Cerasoni

archaeology, photogrammetry, modelling, computational archaeology, digital archaeology, computer graphics

protocol ,

Mar 08, 2022

Mar 09, 2022

Citation: Yu Tang, Jacopo Niccolo Cerasoni, Emily Yuko Hallett High Resolution "DIY" Photogrammetry - 'HRP' Protocol https://dx.doi.org/10.17504/protocols.io.b53xq8pn

This is an open access protocol distributed under the terms of the **Creative Commons Attribution License** (https://creativecommons.org/licenses/by/4.0/), which permits unrestricted use, distribution, and reproduction in any medium,

59223

In the following protocol, typographical emphases and brackets have been used for software and in-text references.
They are: (1) main sub-steps and softwares in bold, e.g. **Add imagery** and **Adobe Photoshop**; (2) "software functions" in "quotation marks", e.g. "Copy correction settings"; (3) <keyboard buttons> in <angle markers>, e.g. <right click> or <Del>.

Hardware

DSLR or mirrorless Camera
Tripods
LED light or flash
Flash Diffuser
TurnTable
Scale
White or black background cloth
Colorchecker (if colour correction is required)
Remote controller (if not using the mobile phone software remote control)

Software *(generally paid softwares are faster and more efficient)*

Photographic Processing Software

Paid software: DxO Photolab/Adobe Photoshop/Adobe Lightroom/CaptureOne
Free software: Darktable/Rawtherapy/Libraw/DCRaw

Photogrammetry Software

Paid software: Realitycapture/Agisoft Metashape
Free software: Meshroom

Note that this protocol is not presenting a novel photogrammetric method. We aimed to create an easy to follow step-by-step protocol which aims to cover the basics for anyone to learn photogrammetry and create high-resolution 3D models independently. For more detailed discussions and guides on academic and computer graphics photogrammetric applications see the following sources:

In English -

1. Photogrammetric Applications for Cultural Heritage
https://historicengland.org.uk/images-books/publications/photogrammetric-applications-for-cultural-heritage/
2. Photogrammetry in Paleontology - A practical guide
https://www.jpaleontologicaltechniques.org/pasta3/JPT%20N12/Pdf/JPT_n012_Jul.pdf
3. A comparison of methods for creating 3D models of obsidian artifacts
https://www.youtube.com/watch?v=g0YaWDrl5qI&ab_channel=RecordingArchaeology
4. Photogrammetry Workflow - Unity
https://unity3d.com/files/solutions/photogrammetry/Unity-Photogrammetry-Workflow_2017-07_v2.pdf
5. Full Photogrammetry Guide for 3D Artists

Citation: Yu Tang, Jacopo Niccolo Cerasoni, Emily Yuko Hallett High Resolution "DIY" Photogrammetry - 'HRP' Protocol
https://dx.doi.org/10.17504/protocols.io.b53xq8pn

This is an open access protocol distributed under the terms of the **Creative Commons Attribution License** (https://creativecommons.org/licenses/by/4.0/), which permits unrestricted use, distribution, and reproduction in any medium,

https://80.lv/articles/full-photogrammetry-guide-for-3d-artists/
6. RealityCapture tutorial: How to create your first 3D model | Part 1-4 (Multilingual Subtitles)
https://www.youtube.com/watch?v=DTJq1Dz0nxY&ab_channel=CapturingReality
7. The Unbearable Lightness of Mystic "Exposure" Triangle
https://www.fastrawviewer.com/blog/mystic-exposure-triangle
8. New Old Approach to Dynamic Range
https://www.fastrawviewer.com/blog/determining-practical-dynamic-range

In Japanese [日本語で] -
9. 3D Measurement of Lithic Artifacts, Towards Publication and Sharing of Results. [石器の3D計測、成果の公開・共有　を目指して] - Written by Atsushi Noguchi

http://54.64.145.19/dspace/bitstream/11177/7014/1/data_science_salon01_009_014.pdf
10. 3D Technology and Archaeology [3D技術と考古学] - Book edited by Satoru Nakazono
https://www.yuzankaku.co.jp/products/detail.php?product_id=8377
https://www.yuzankaku.co.jp/user_data/special_3d.php *link to the stone tools models presented in the book*

Both academic and computer graphics fields apply photogrammetric processes for different reasons. Each field has its own advantages and disadvantages. For example, heritage preservation specialists rarely discuss the principles of computer graphics (CG), while the CG experts rarely discusses how to optimize the accuracy of scanning. Here we presented both sides into one single overarching method.

If you wish to use this method for scientific purposes, please remember to **record your metadata and workflow**. Make sure to keep notes on (1) number of photographs taken, (2) camera settings, (3) software functions/settings, and any other information which will be useful at a later date.

This will allow you to be able to produce and write documents and methodological texts at the highest possible degree of precision, making sure to not forget or ignore any useful piece of information.

Please note that some of the softwares used in this protocol require specific minimum configurations to run properly. Before using any of the softwares present here, check that your computer system meets the minimum configuration requirements.

Example: RealityCapture requires an Nvidia graphics card to generate meshes and textures.
RealityCapture runs on 64-bit machines with **at least 8GB of RAM**, 64bit Microsoft Windows 7/8/8.1/10, using a graphics card with an **nVidia** CUDA 2.0+ GPU and at least 1 GB of RAM.

Citation: Yu Tang, Jacopo Niccolo Cerasoni, Emily Yuko Hallett High Resolution "DIY" Photogrammetry - 'HRP' Protocol https://dx.doi.org/10.17504/protocols.io.b53xq8pn

This is an open access protocol distributed under the terms of the **Creative Commons Attribution License** (https://creativecommons.org/licenses/by/4.0/), which permits unrestricted use, distribution, and reproduction in any medium,

Setting Photographic Environment

1 Collect and prepare equipment.

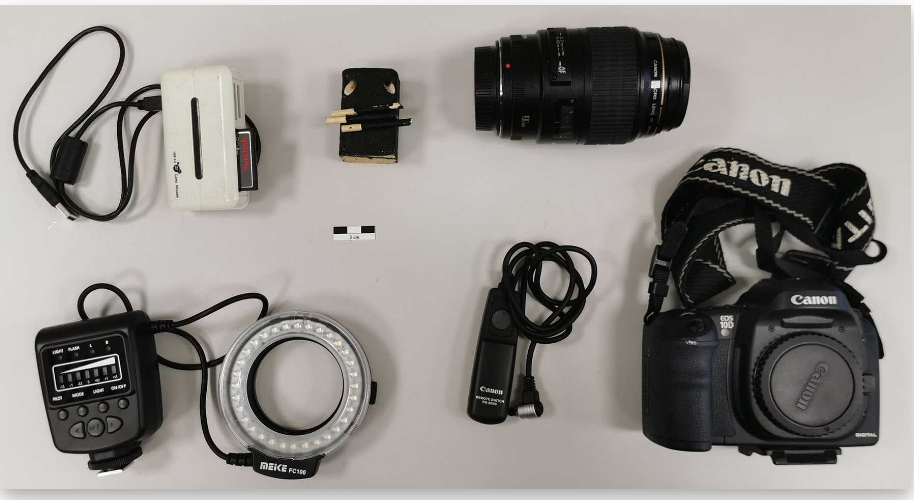

See Materials section for full list of equipment used in this protocol.

Generally, the use digital cameras, light boxes, and a turntable for photography are preferred. Nevertheless, in recent years the quality of cell phone photos has increased, and there are several Photogrammetry softwares on iPhone systems that can be used to scan easily. However, due to the limited image quality, scanning with mobile phones is not suitable for accurate scanning that requires less than millimeter accuracy.

⚠ If using a mobile phone, scans will never be as accurate and precise and they could be when using a digital camera.

Although it is possible to take excellent quality scans with a mobile phone, several considerations and extra steps are necessary to achieve this. As mobile phones to not usually have manual control for photography, extra-party softwares are required. Furthermore, photographs should be taken in RAW (DNG) format, using a remote control and adequate lighting.

Furthermore. due to the small sensor size of mobile phone cameras, it is often necessary to take more pictures or to get closer to the object to achieve high-detail models. Although this is not problematic when wanting to apply this method to simple objects, complex or more detail objects will be extremely difficult to correctly photograph. In turn, DSLR or mirrorless camera will give better results with less time and effort.

Citation: Yu Tang, Jacopo Niccolo Cerasoni, Emily Yuko Hallett High Resolution "DIY" Photogrammetry - 'HRP' Protocol https://dx.doi.org/10.17504/protocols.io.b53xq8pn

This is an open access protocol distributed under the terms of the **Creative Commons Attribution License** (https://creativecommons.org/licenses/by/4.0/), which permits unrestricted use, distribution, and reproduction in any medium,

2 Set the camera on a tripod.

Please note that the camera support greatly depends on the surroundings where the photographic station has been set up. Tripods work best on open spaces. On tighter spaces, photography arms with a clamp to attach to stable surfaces (such as tables) are more suitable.

2.1 Choose a solid color for the background if possible (Black or white is recommended).

A solid color for the background will aid the subsequent processes in a variety of ways. They are:

1. Help separating the photographed object from the background.
2. The photogrammetric software will hardly detect any feature or key point from the background, therefore making the automatic matching process easier.
3. A solid color will be easier to mask on the photogrammetric software.

Furthermore, contrast between the object and the background, set by adjusting the lighting, will aid in the isolation of the photographed object from the background. For further explanations on the process of background, lighting and contrast selection see *Step 3* of the 'SOAP' protocol (dx.doi.org/10.17504/protocols.io.b53zq8p6)

3 Set the turntable on the table and set the items you want to scan on it, placing it as central as possible.

It is recommended to use the remote control shutter with between 2 to 10 second timer to avoid shake and bluriness. If the camera does not include one, remote control can be accessed via WiFi or Bluetooth; both Android and iOS universal applications exist for this purpose.

Setting Lighting Environment

Citation: Yu Tang, Jacopo Niccolo Cerasoni, Emily Yuko Hallett High Resolution "DIY" Photogrammetry - 'HRP' Protocol https://dx.doi.org/10.17504/protocols.io.b53xq8pn

This is an open access protocol distributed under the terms of the **Creative Commons Attribution License** (https://creativecommons.org/licenses/by/4.0/), which permits unrestricted use, distribution, and reproduction in any medium,

4 If using a turntable, lighting for photogrammetry does not need to be completely uniform (360 degrees), but you need to keep the front lighting as uniform as possible. Diffused lighting is also recommended.

Ring lights are a good choice, but you can also choose 2 or more LED lights set on the left and right sides of the camera.

> Ring flashes (such as the Godox Witstro AR400) will give efficient and bright lighting, however are somewhat expensive. LED lights are not bright enough however they are very cheap.

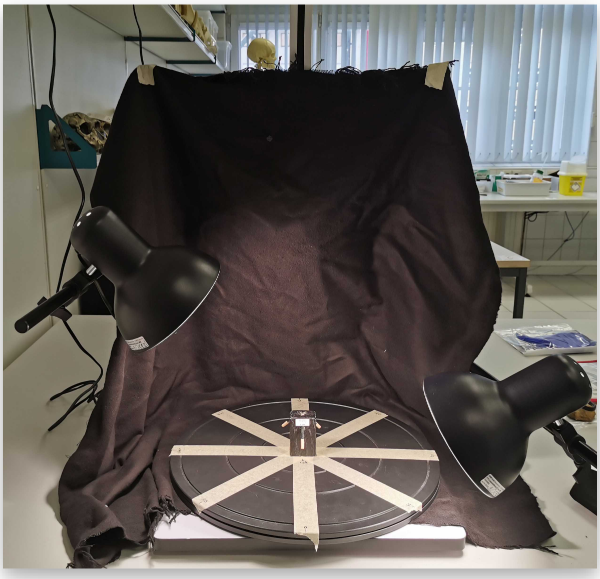

Example of front, diffused lighting.

Photographic Reference

5 The following steps (6-14) present photographic methods and theories which are fundamental for the correct capturing of photographs for photogrammetric purposes. Further detailed explanation for all the discussed techniques and functions can by consulting the "Small Object and Artefacts Photography" protocol (dx.doi.org/10.17504/protocols.io.b53zq8p6).

Camera Settings

6 RAW format (CR2, CRW, NEF, PEF...) is usually preferred for photogrammetry, this will provide a wider color gamut and more correct color calibration in post-processing.

> If high resolution of color and final model is not of importance, JPG/JPEG formats can also be used resulting in a more convenient and faster option.

Citation: Yu Tang, Jacopo Niccolo Cerasoni, Emily Yuko Hallett High Resolution "DIY" Photogrammetry - 'HRP' Protocol https://dx.doi.org/10.17504/protocols.io.b53xq8pn

This is an open access protocol distributed under the terms of the **Creative Commons Attribution License** (https://creativecommons.org/licenses/by/4.0/), which permits unrestricted use, distribution, and reproduction in any medium,

7 **Manual mode** is usually selected because we want to have no difference in brightness during photography.

Changing the brightness does not change the model generation results to a certain extent, however, it directly affects the texture generation. For some items with extreme contrast, brightness can be changed at times during the process, but will require some practice to use this effectively.

Manual mode is always suggested to use for photogrammetric purposes.

8 **Aperture.**

Regular Photogrammetry usually selects an aperture between F/5.6 and F/16. Different aperture sizes depend on the focal length and the size of the subject. Too small aperture will cause diffraction and reduce the quality of the photo, while too big aperture will reduce the depth of field.

Generally, for small objects an aperture of F/11 is suggested. Higher apertures such as F/16 are necessary in some cases if the object is too small, however this requires a high-quality lens (e.g. SEL90M28G) so to avoid diffraction.

Depth of field can be calculated according to the set aperture. Depth of field can be calculated from the focal length, distance to subject, the acceptable circle of confusion size and aperture .

The object should always be placed in the **middle of the camera frame**, which indirectly determines the distance from the camera to the object, as well as the depth of field.

9 **Exposure.** Setting exposure time and ISO

First set ISO to an optional value such as 200 (high ISO values will result in noisy images). Call up the histogram in the camera and try to shoot the object. Adjust the exposure time to the appropriate time so that the vertical part of the histogram representing the subject is in the middle and the highlighted part of the histogram.

If you are using LED lighting, the exposure time will usually be very long at this time. Reduce the exposure time and or you can increase the ISO value slightly. Until the ISO does not cause too much noise in your photos and the exposure time is at an acceptable value (too

Citation: Yu Tang, Jacopo Niccolo Cerasoni, Emily Yuko Hallett High Resolution "DIY" Photogrammetry - 'HRP' Protocol https://dx.doi.org/10.17504/protocols.io.b53xq8pn

This is an open access protocol distributed under the terms of the **Creative Commons Attribution License** (https://creativecommons.org/licenses/by/4.0/), which permits unrestricted use, distribution, and reproduction in any medium,

long an exposure time can make the photography process too long).

If you use flash light, this adjustment will become easier.
However, I usually recommend using at least ISO200 instead of ISO100, because ISO200 does not cause much noise and will provide a shutter speed of one stop.

If the photo is very dark, a lower ISO will not make the overall noise less, as the noise in the dark areas will be amplified when the exposure is increased in post-processing.

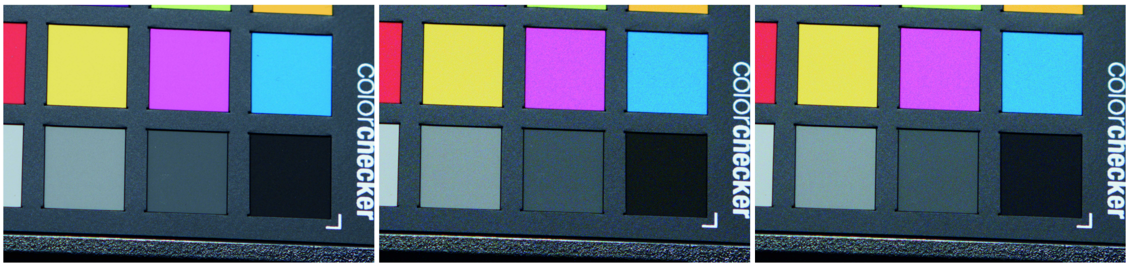

Sample images with different shutter and ISO values
Left - Aperture f/2.5, shutter 1/3s, **ISO 100**, -0.33 EV exposure compensation
Center - Aperture f/2.5, shutter 1/60s, **ISO 100**, 4 EV exposure compensation
Right - Aperture f/2.5, shutter 1/60s, **ISO 1600**

9.1 Advanced methods of measuring exposure.

As the in camera histogram shows a preview image in JPG format, exposure should be directly evaluted on the RAW format photograph.

After taking a photograph, decoduing of the RAW photo to check the photograph's exposure is required.

To do so, Rawdigger can be used. To open a RAW photo on Rawdigger, hold down SHIFT to frame the object. Select
on selection at the top left to select the histogram.

Citation: Yu Tang, Jacopo Niccolo Cerasoni, Emily Yuko Hallett High Resolution "DIY" Photogrammetry - 'HRP' Protocol https://dx.doi.org/10.17504/protocols.io.b53xq8pn

This is an open access protocol distributed under the terms of the **Creative Commons Attribution License** (https://creativecommons.org/licenses/by/4.0/), which permits unrestricted use, distribution, and reproduction in any medium,

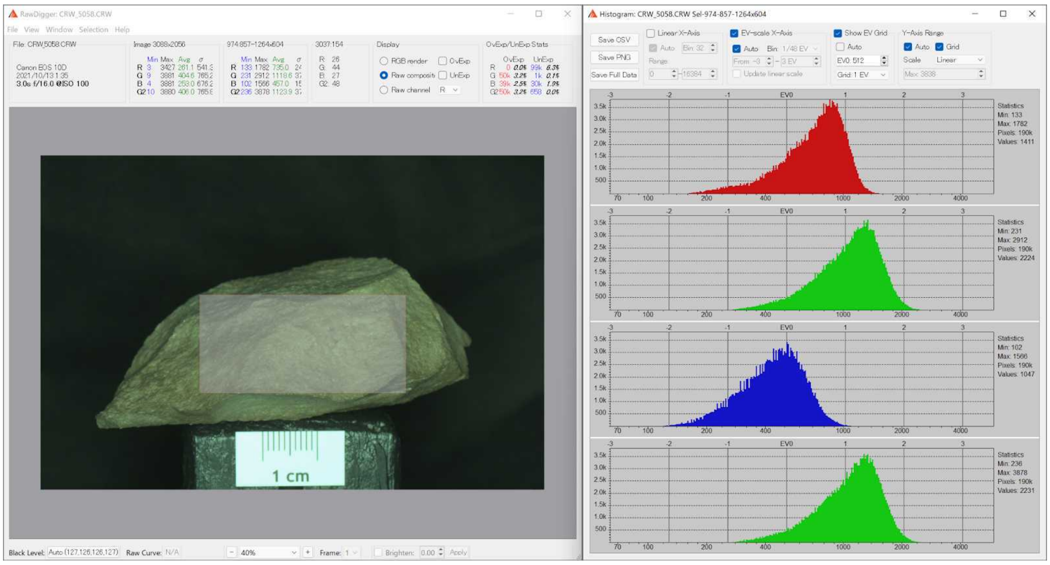

In this photo, the stone artefact is basically above EV-0, making it correctly exposed.

The following exposure method was developed following enhancement of the methods explicated in sources 7 and 8 (before start section).

If you are interested in the correct representation of the colours in the final model, Color Checker can be used, making sure that both colorchecker and object are correctly exposed. (Or adjust the exposure multiplier by calculation, which is slightly more complicated).

It is important to note that the method we will describe for processing photos does not correct for color. In the case where perfect color wants to be represented, Cross-Polarization needs to be adjusted, followed by very complex correction in post-processing. These steps are not explicated in this protocol, as perfect color representation falls outside the scope of this method, and it will add further steps to the protocol.

10 **Set white balance.**

You can set an arbitrary white balance when taking pictures and adjust it in post-processing. If possible, use a gray card or color checker and adjust the white balance based on it in post-processing.

Photography

11 **METHOD 1 -** If the object can be placed on a turntable, place the object on it and take a photograph inbetween slight rotations of the turntable (e.g. 15° or 30°). Repeat the process until

Citation: Yu Tang, Jacopo Niccolo Cerasoni, Emily Yuko Hallett High Resolution "DIY" Photogrammetry - 'HRP' Protocol https://dx.doi.org/10.17504/protocols.io.b53xq8pn

This is an open access protocol distributed under the terms of the **Creative Commons Attribution License** (https://creativecommons.org/licenses/by/4.0/), which permits unrestricted use, distribution, and reproduction in any medium,

the object ends in its original position (360°).

> Ideally, more rotations are photographs of the object the better it will be. The rotations should be envisioned as a full equally distanced "sphere" of photographs around the object. Consider 5 as a minumum number of rotations for a high quality final model.

11.1 Change the placement of the object onto the turntable to so show faces of the object which were not previously in frame (visible through the camera). Repeat step 11 for each placement of the object until you have photograph every face or side.

12 **METHOD 2 -** If the object can't be placed properly onto different faces, change the height and position of the camera so to frame the object at different angles and take photographs in a circular motion. Repeat this action making sure to photograph the object's upper and lower face.

12.1 When changing the height of the camera, it can also be moved closer to the object. This will make the distance between the camera and the object consistent.

Citation: Yu Tang, Jacopo Niccolo Cerasoni, Emily Yuko Hallett High Resolution "DIY" Photogrammetry - 'HRP' Protocol https://dx.doi.org/10.17504/protocols.io.b53xq8pn

This is an open access protocol distributed under the terms of the **Creative Commons Attribution License** (https://creativecommons.org/licenses/by/4.0/), which permits unrestricted use, distribution, and reproduction in any medium,

12.2

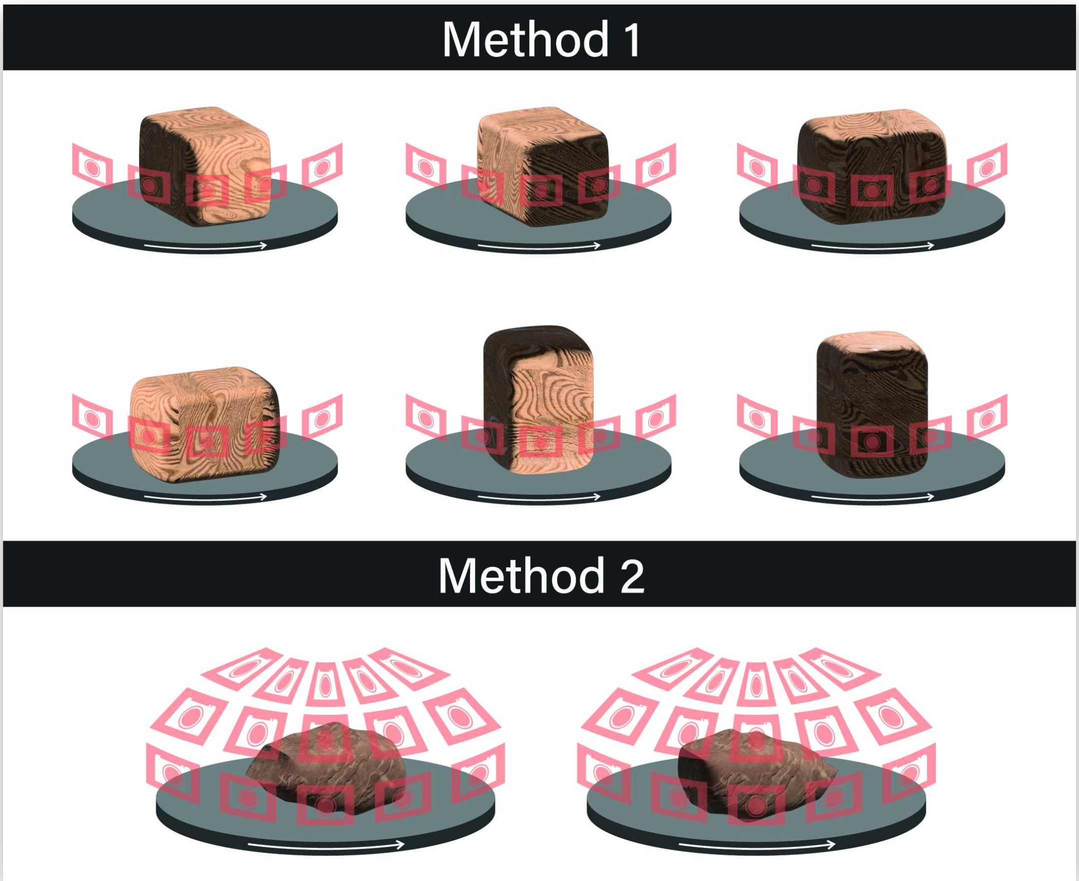

Visual representation of Method 1 and 2 explained in *Step 12*. In grey the rotating turntable is shown. In red are the virtual positions of the camera lens, showing the variation in height at constant distance for each of the two methods. Note that Method 1 is used for an object than can be placed comfortably on different faces, while Method 2 is used for an object which can be placed without the aid of external holders only on two separate faces.

13 Place the object on the turntable or holder, make sure it is centered on the turntable. The whole object should always be within the frame of the camera.

Press the shutter by remote control and rotate the turntable. Repeat this operation until it rotates one circle. The angle of rotation can be smaller depending on the details of the subject. Ideally, a minimum of 18 times per rotation is preferred.

Citation: Yu Tang, Jacopo Niccolo Cerasoni, Emily Yuko Hallett High Resolution "DIY" Photogrammetry - 'HRP' Protocol https://dx.doi.org/10.17504/protocols.io.b53xq8pn

This is an open access protocol distributed under the terms of the **Creative Commons Attribution License** (https://creativecommons.org/licenses/by/4.0/), which permits unrestricted use, distribution, and reproduction in any medium,

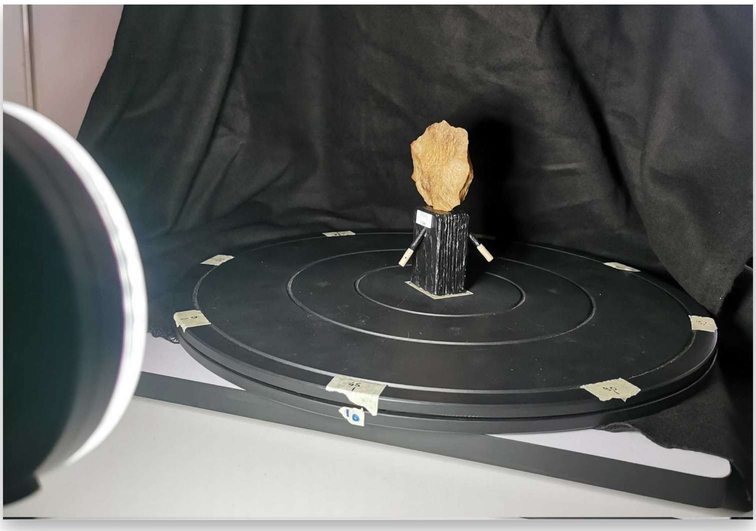

Example of lithic artefact on holder. Note the marking every 45° on the base, with relevant 0° point at the base of the turntable.

It is essential to include a scale in the photographs of the selected object. Ideally, the scale should be fully visible in at least 4 or 5 of the photos so to aid in the alignment of all the photographs.

If the object cannot be rotated, the object can also be placed in the middle of a table, with the photographed rotating and scanning 360° around the object. This requires a strong light source, and it is suggested to use a strobe mounted on the camera. This method is commonly used and it will provide a very quick photo process. However, because it is done manually by a person, there will be quality deviations between photos.

14 General photography methods and ideas.

Because the basic way of photogrammetry's three-dimensional reconstruction is parallax, we need to take at least 2 photos of each surface of the subject. Therefore, before beginning the process, consider the following when photographing:

1. Imagine a regular polyhedron (e.g., a regular dodecahedron) and place the subject in its center. Subdivide the polyhedron so that each vertex of the subdivision will be the ideal camera position, with each camera facing the center.

2. Imagine a simplified object, place the camera vertically facing all the simplified faces, and all the edges for the shot.

Citation: Yu Tang, Jacopo Niccolo Cerasoni, Emily Yuko Hallett High Resolution "DIY" Photogrammetry - 'HRP' Protocol https://dx.doi.org/10.17504/protocols.io.b53xq8pn

This is an open access protocol distributed under the terms of the **Creative Commons Attribution License** (https://creativecommons.org/licenses/by/4.0/), which permits unrestricted use, distribution, and reproduction in any medium,

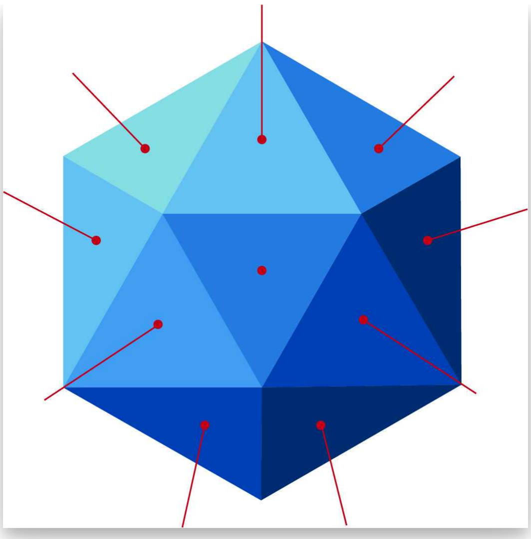

Example of dodecahedronal perspective. Object (set in the middle) is photographed from every face of the imaginary dedecahedron. Red dots are the points that the camera focuses on, red lines are the directions the camera faces.

14.1 If the object is particularly reflective or shiny (e.g. obsidian), dusting or matting of the surface will be required. For more details, see video n. 3 in the "Before Start" section.

Importing Photographs - RAW Processing

15 Open DxO Photolab (image processing software). You can also choose Adobe Photoshop, Adobe Lightroom or Capture One.

When processing RAW photographs, LibRaw can also be used, which is completely free and commonly used. However note that this software but requires some programming skills to make it work automatically with a large number of photos. It also requires post color calibration.

Please note that the processing of RAW images is optional. Although the following steps (*15-25*) will result in the best possible outcome, the use of unprocessed RAW images will still produce a good final result.

16 In the Photo Library, select the folder where the Raw format images are located. Double-click on any image to enter customize mode.

Citation: Yu Tang, Jacopo Niccolo Cerasoni, Emily Yuko Hallett High Resolution "DIY" Photogrammetry - 'HRP' Protocol https://dx.doi.org/10.17504/protocols.io.b53xq8pn

This is an open access protocol distributed under the terms of the **Creative Commons Attribution License** (https://creativecommons.org/licenses/by/4.0/), which permits unrestricted use, distribution, and reproduction in any medium,

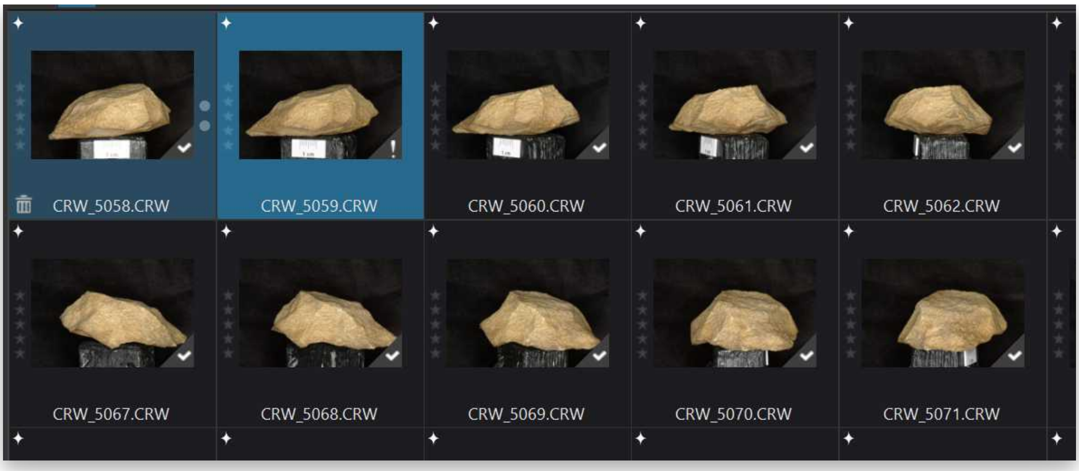

17 Increase Microcontrast.

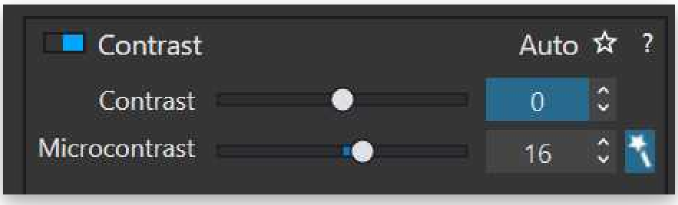

18 Decrease Highlights, Midtones.

Increase Shadows, Blacks,

If necessary, adjust the exposure.

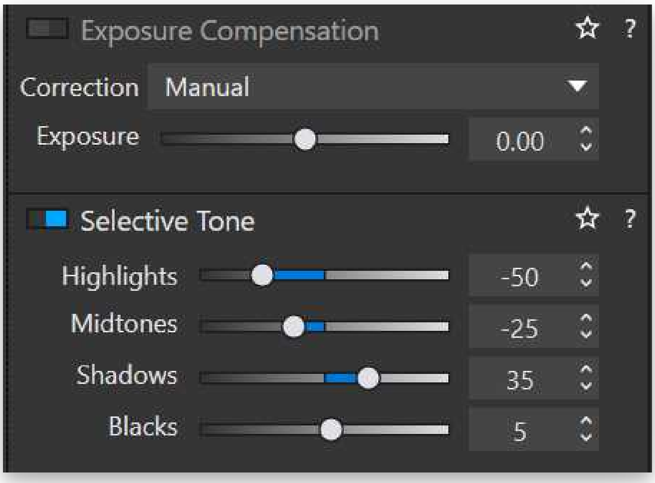

Values can differ depending on the situation.
The purpose is to recover details from highlights and shadows, and to make Micro detail

Citation: Yu Tang, Jacopo Niccolo Cerasoni, Emily Yuko Hallett High Resolution "DIY" Photogrammetry - 'HRP' Protocol https://dx.doi.org/10.17504/protocols.io.b53xq8pn

This is an open access protocol distributed under the terms of the **Creative Commons Attribution License** (https://creativecommons.org/licenses/by/4.0/), which permits unrestricted use, distribution, and reproduction in any medium,

obvious.

Please note that the following method describes a non-linear colour adjustment of pixels within a photograph. Studies have shown that this method can at times be non-ideal for scientific purposes (Rossner and Yamada 2004; https://doi.org/10.1083/jcb.200406019). Alternatively, photo filters can be used to achieve a linear adjustment, although they require subjective judgement.

If the method described here is used for scientific publication, we suggest disclosing that this manipulation technique was used.

19 Set a value for White Balance.You can use the Pick Color tool to take the values from the gray card or from the neutral gray part.

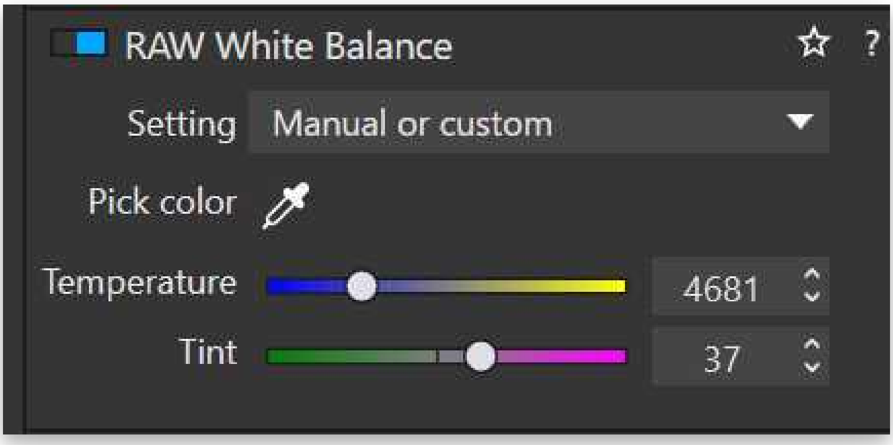

20 Turn on "Denoising", if the ISO is not very high then the default value is usually very suitable.

Citation: Yu Tang, Jacopo Niccolo Cerasoni, Emily Yuko Hallett High Resolution "DIY" Photogrammetry - 'HRP' Protocol https://dx.doi.org/10.17504/protocols.io.b53xq8pn

This is an open access protocol distributed under the terms of the **Creative Commons Attribution License** (https://creativecommons.org/licenses/by/4.0/), which permits unrestricted use, distribution, and reproduction in any medium,

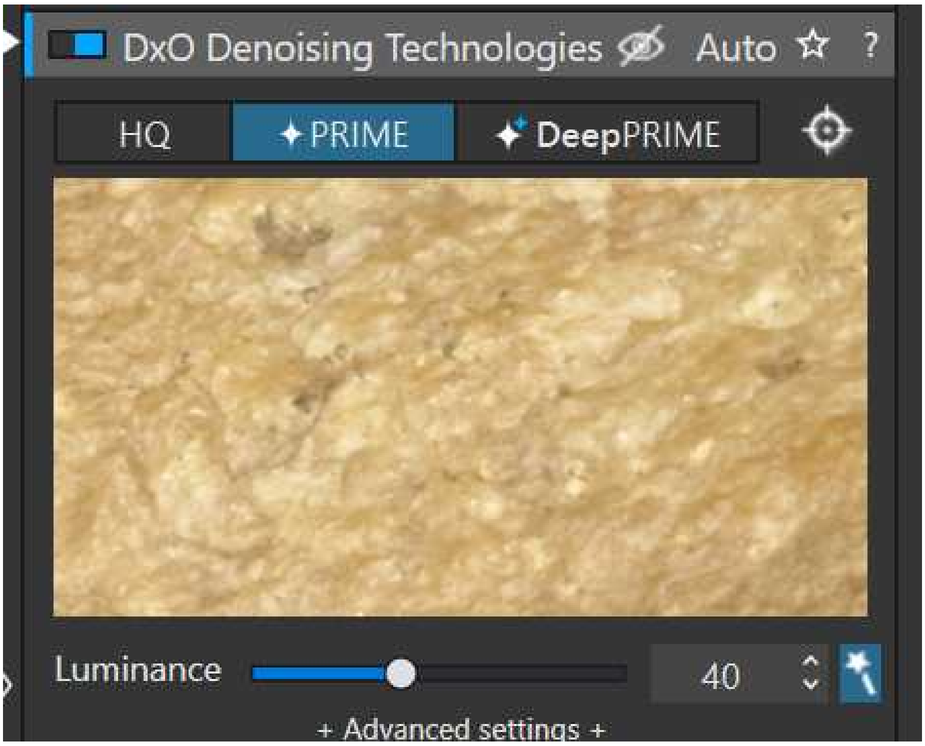

21 Right-click on the photo and select "Copy correction settings" (<Ctrl+Shift+C>).

22 Press <Ctrl+A> at the same time to select all photos, right-click on the photo list below and select "Paste all correction settings" (<Crtl+Shift+V>).

23 Select "Export to Disk" at the bottom right of the screen to export the corrected photos. Usually JPEG is enough, but select 100 for Quality to get good quality.

24 Select a Path and export.

Citation: Yu Tang, Jacopo Niccolo Cerasoni, Emily Yuko Hallett High Resolution "DIY" Photogrammetry - 'HRP' Protocol https://dx.doi.org/10.17504/protocols.io.b53xq8pn

This is an open access protocol distributed under the terms of the **Creative Commons Attribution License** (https://creativecommons.org/licenses/by/4.0/), which permits unrestricted use, distribution, and reproduction in any medium,

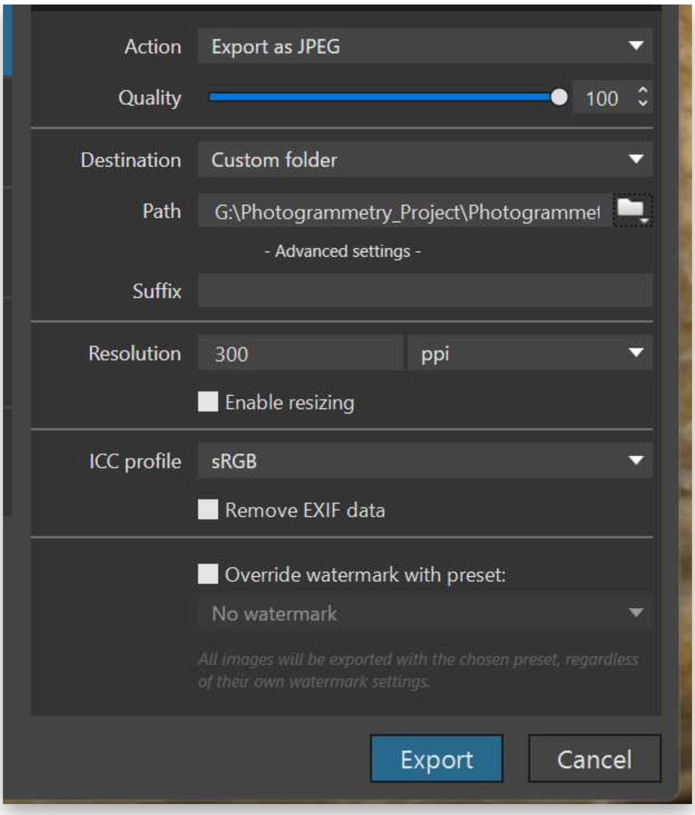

25 In general, photos used for photogrammetry do not require any other processing, and it is important to note that, no correction to lens distortion should be applied. This is because the distortion correction needs to be calculated together with the parallax calculation in the photogrammetry software.

Photogrammetry Software Post-Processing

26 **Open RealityCapture.**

RealityCapture is a photogrammetry software that can generate 3D models from photographs or laser scans without seams. It has several payment options and a free version is available for educational institutions.

> Other softwares such as Agisoft Metashape or 3DF Zephyr can also be used. They are both extremely efficient and usable softwares, with lower requirements of CPU memory, and being able to create 3D meshes with much lower CPU power. However, in both cases a higher degree of post-processing will be required as they tend to produce higher noise during the production of 3D models compared to RealityCapture.

27 **Add imagery.**

Drag and drop all photos or the folder to the software. Then you can see that all the pictures have

Citation: Yu Tang, Jacopo Niccolo Cerasoni, Emily Yuko Hallett High Resolution "DIY" Photogrammetry - 'HRP' Protocol https://dx.doi.org/10.17504/protocols.io.b53xq8pn

This is an open access protocol distributed under the terms of the **Creative Commons Attribution License** (https://creativecommons.org/licenses/by/4.0/), which permits unrestricted use, distribution, and reproduction in any medium,

been added to the software.

27.1 If you use the same focal length, select on "Images" and then "Group button" at the bottom. Following this, all photos will be aligned with the same internal parameters.

27.2 select the "ALIGNMENT TAB" and select "Settings" to see the alignment settings. In most cases, the default settings are fine.

27.3 Generally, you just need to adjust the Image overlap to "Low" and select "Detector Sensitivity" depending on the surface texture of the object.

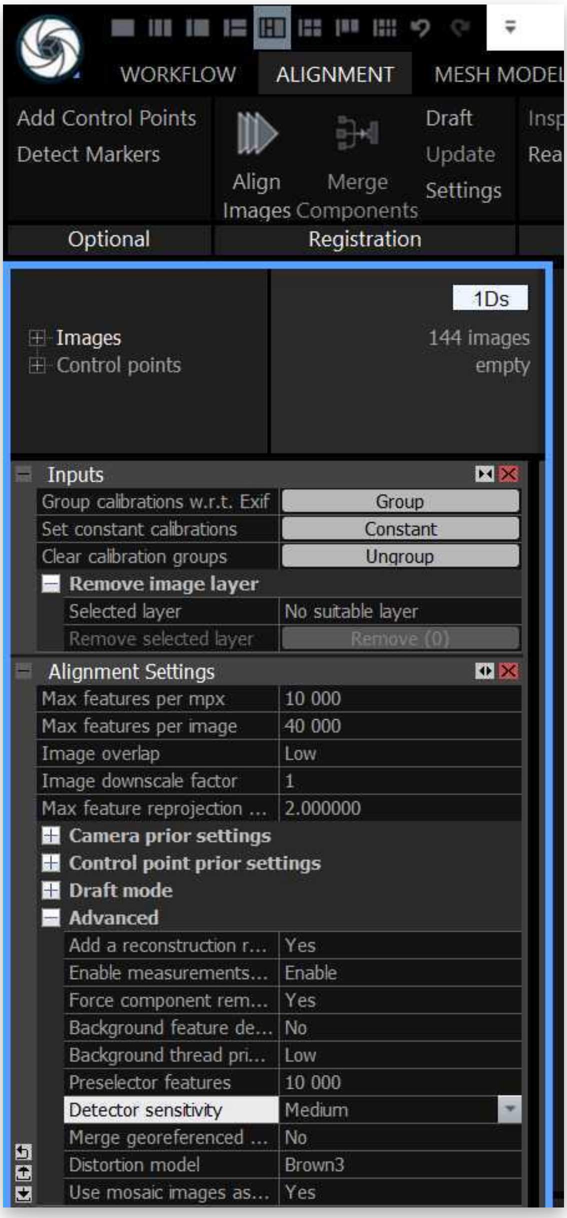

Citation: Yu Tang, Jacopo Niccolo Cerasoni, Emily Yuko Hallett High Resolution "DIY" Photogrammetry - 'HRP' Protocol https://dx.doi.org/10.17504/protocols.io.b53xq8pn

This is an open access protocol distributed under the terms of the **Creative Commons Attribution License** (https://creativecommons.org/licenses/by/4.0/), which permits unrestricted use, distribution, and reproduction in any medium,

28 Align Images.

Select "Align Images". If there are no problems with the photos, almost all of them will be aligned into one Component. If the alignment is not successful at once, you can select "Align Images" to align the remaining photos manually.

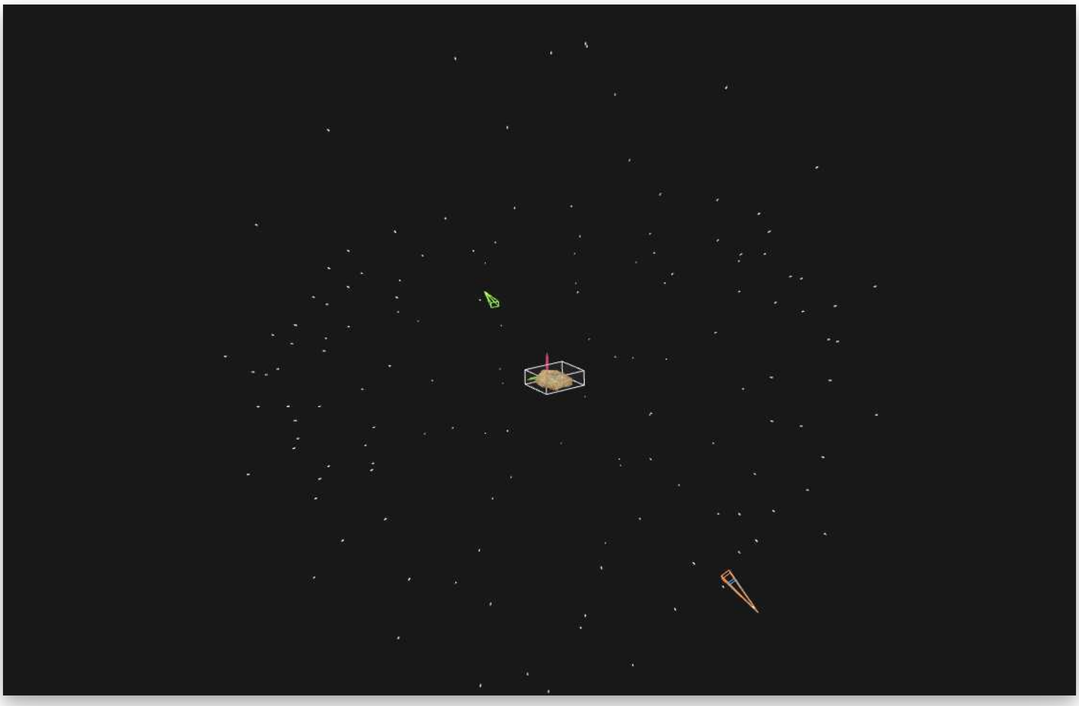

28.1 To note, alignment is not always successful at the first try. When it fails, it is suggested to repeatedly attempt the allignment and practice. Experience and time spent practicing are key components to the successful allignment of complex objects.

29 Reconstruction.

Select "MESH MODEL TAB" and select "Set Reconstruction Region" to define a region you want to reconstruct, you can also modify the region manually in the "3Ds" window.

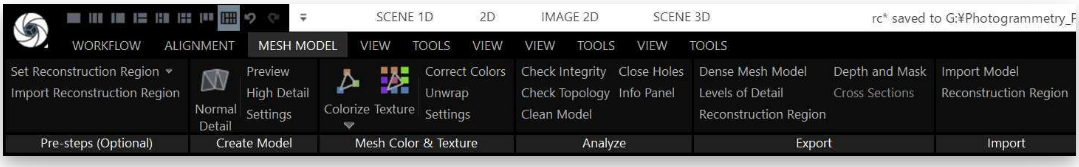

29.1 Select "Normal Detail" to reconstruct the model. You can also select "High Detail" to get maximum detail if you are using a digital camera and the ISO is not too high.

Citation: Yu Tang, Jacopo Niccolo Cerasoni, Emily Yuko Hallett High Resolution "DIY" Photogrammetry - 'HRP' Protocol https://dx.doi.org/10.17504/protocols.io.b53xq8pn

This is an open access protocol distributed under the terms of the **Creative Commons Attribution License** (https://creativecommons.org/licenses/by/4.0/), which permits unrestricted use, distribution, and reproduction in any medium,

29.2

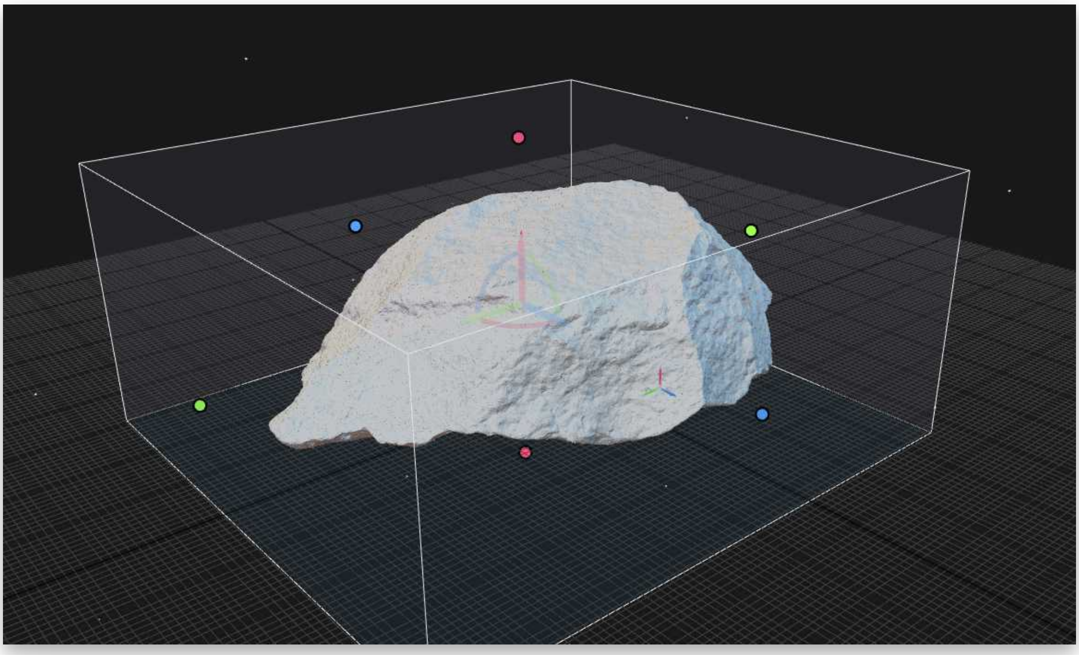

Reconstruction result.

30 Simplification Model.

If the model has too many faces, you may get a message when the reconstruction is completed that Warning "Not enough video memory", however, this is just a display issue. Next, simplication of the model can be applied to make it more manageable and easier to view.

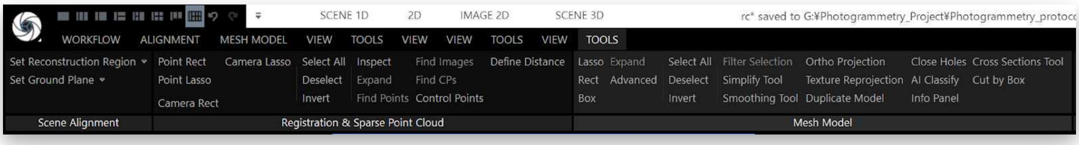

Faces are simply the individual polygons which, when put together, form the surface of the 3D model.

Generally, for online viewing of 3D models a maximum of 20,000 t0 30,000 faces, or polygons, should be considered.

In this case, given the scientific and measurement purposes of the developed model, several millions of faces can be reached.

30.1 Select "Simplify Tool" in the "TOOL TAB", it is recommended to select type to "Relative" and set the value to 25 or 50. The Relative method will preserve the

Citation: Yu Tang, Jacopo Niccolo Cerasoni, Emily Yuko Hallett High Resolution "DIY" Photogrammetry - 'HRP' Protocol https://dx.doi.org/10.17504/protocols.io.b53xq8pn

This is an open access protocol distributed under the terms of the **Creative Commons Attribution License** (https://creativecommons.org/licenses/by/4.0/), which permits unrestricted use, distribution, and reproduction in any medium,

maximum details.

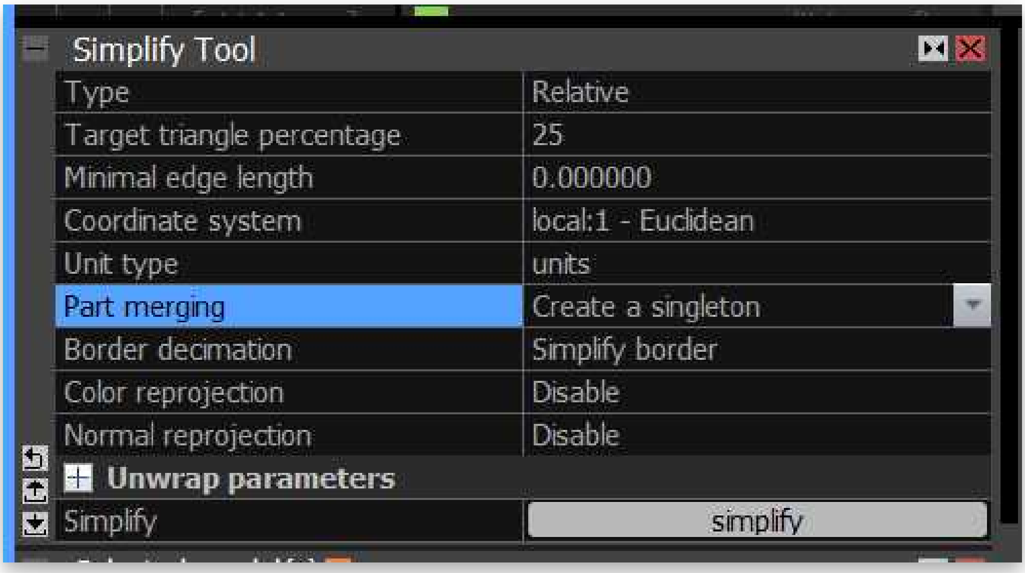

30.2 You can also choose "Part Merging" as "Create a singleton" (if the model is not very large).

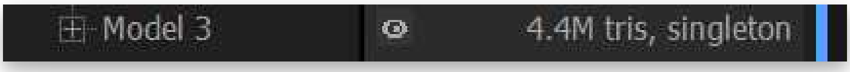

31 **Generate a texture.**

Select "MESH MODEL TAB" and <click> on "Color and Texture Settings".

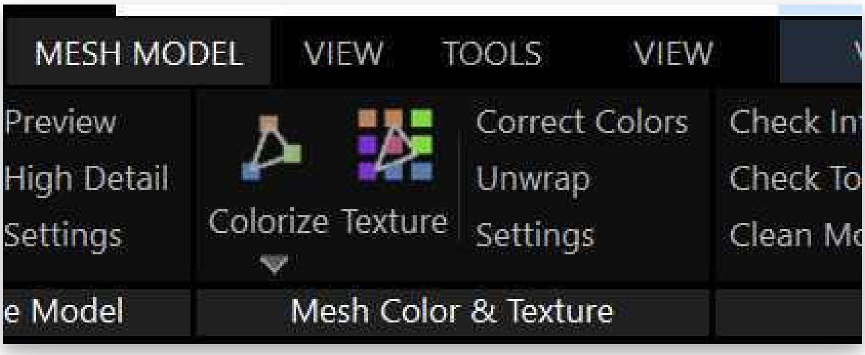

3D Texturing is the creation of a 2D image (texture map) by wrapping it a around a 3D object and defining how light would affect it.

Texture maps can be projected onto a 3D object allowing to look at a previously blank model with a texture, or projected image onto it.

Citation: Yu Tang, Jacopo Niccolo Cerasoni, Emily Yuko Hallett High Resolution "DIY" Photogrammetry - 'HRP' Protocol https://dx.doi.org/10.17504/protocols.io.b53xq8pn

This is an open access protocol distributed under the terms of the **Creative Commons Attribution License** (https://creativecommons.org/licenses/by/4.0/), which permits unrestricted use, distribution, and reproduction in any medium,

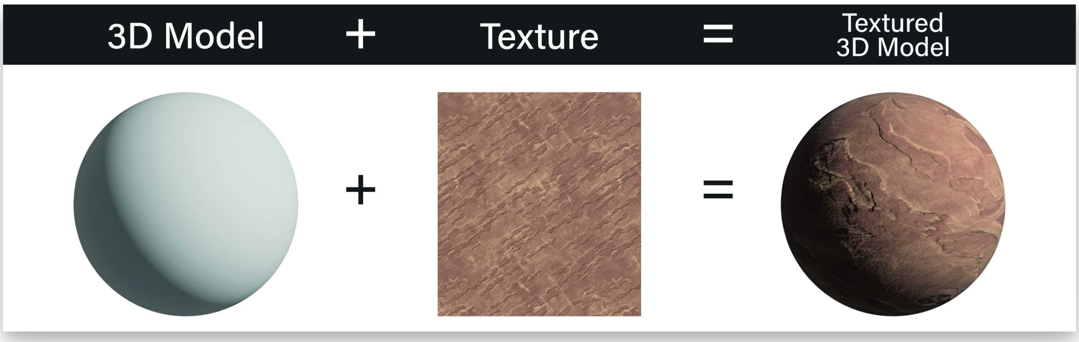

Visual explanation of how textures can be mapped on 3D model to created textured 3D models.

31.1 Usually the default settings of 8K texture and other settings are almost enough. If you don't need such a large map, you can also choose 4196 or 2048.

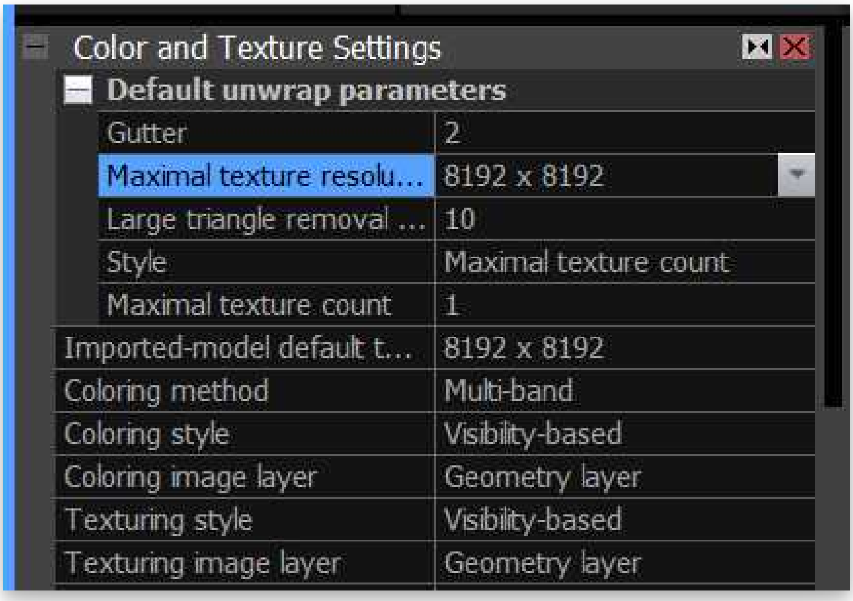

31.2 Generate texture.

Citation: Yu Tang, Jacopo Niccolo Cerasoni, Emily Yuko Hallett High Resolution "DIY" Photogrammetry - 'HRP' Protocol
https://dx.doi.org/10.17504/protocols.io.b53xq8pn

This is an open access protocol distributed under the terms of the **Creative Commons Attribution License** (https://creativecommons.org/licenses/by/4.0/), which permits unrestricted use, distribution, and reproduction in any medium,

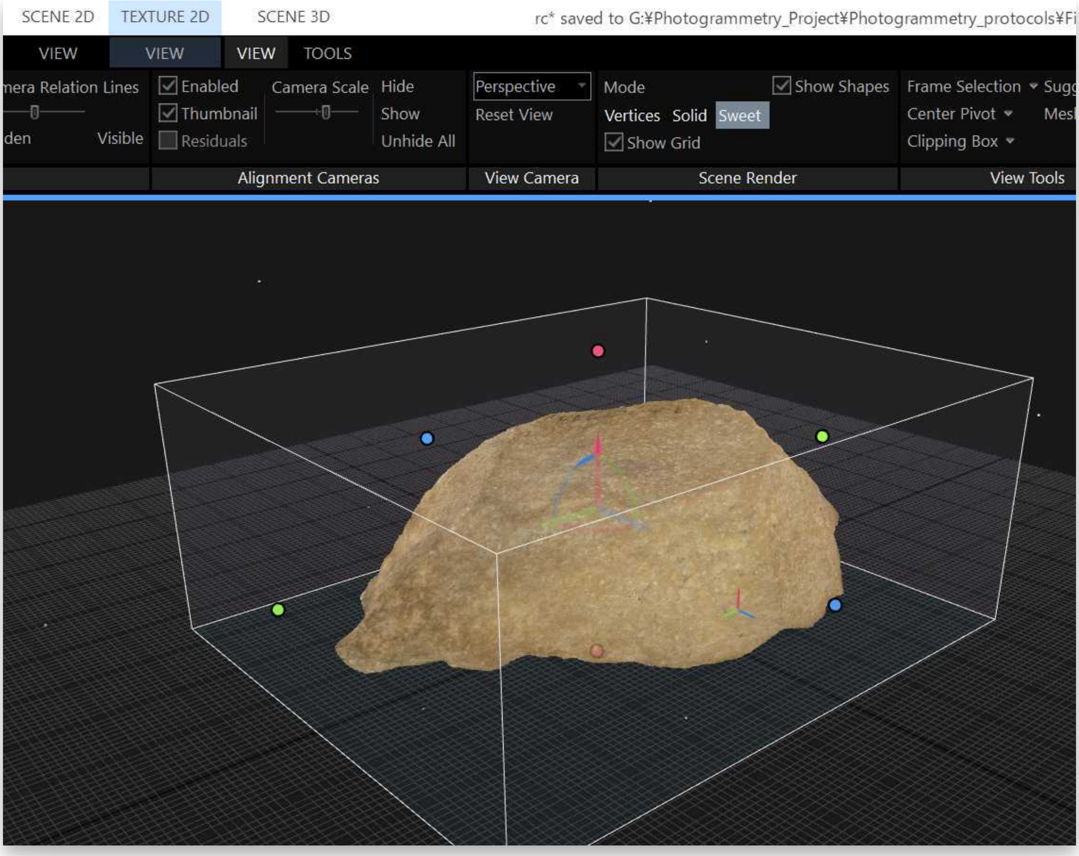

32 Define scale.

Select "ALIGNMENT TAB" and select "Control Points".

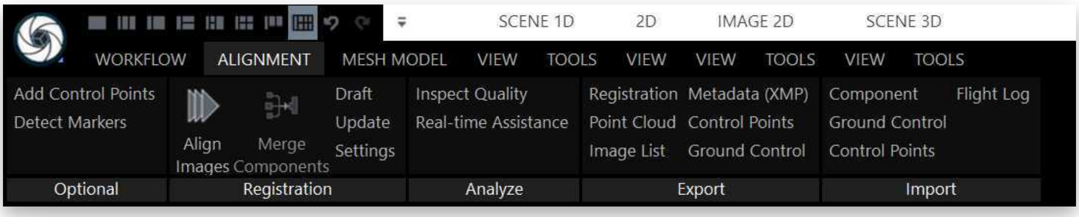

32.1 Select 2 separate photos. Click on at least 2 points, Point 0 and Point 1, on each of the 2 sets of photos.

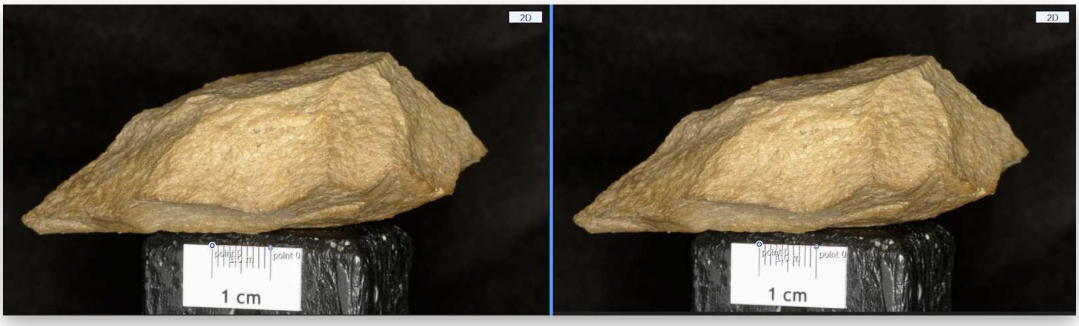

Citation: Yu Tang, Jacopo Niccolo Cerasoni, Emily Yuko Hallett High Resolution "DIY" Photogrammetry - 'HRP' Protocol https://dx.doi.org/10.17504/protocols.io.b53xq8pn

This is an open access protocol distributed under the terms of the **Creative Commons Attribution License** (https://creativecommons.org/licenses/by/4.0/), which permits unrestricted use, distribution, and reproduction in any medium,

32.2 After adding "Control Point", click the green plus symbol in "1Ds" to activate the point and then check the accuracy of the point. If the accuracy is less than the expected value such as 1px, the point is not misplaced.

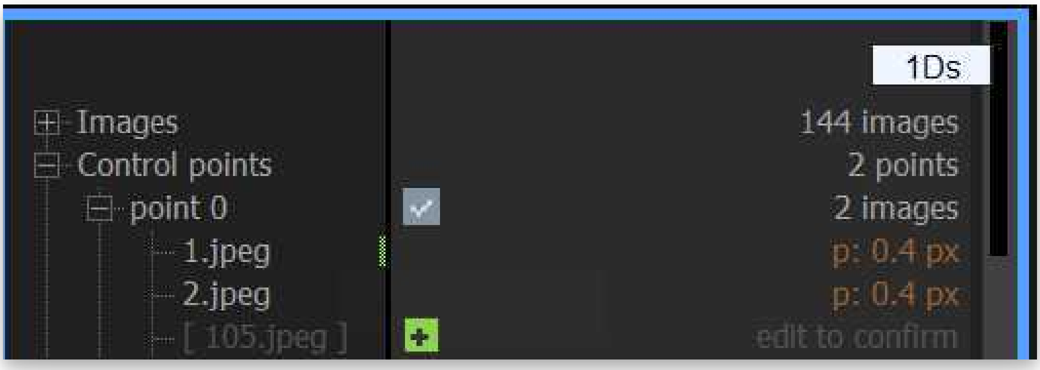

32.3 Select "Create distance" in the "1Ds" window and select distance 0 to change the names of the two points A and B to Point 0 and Point 1.

32.4 Set the Defined distance to the real distance.

32.5 Click "Update" in the "ALIGNMENT TAB" to update to the actual size.

Citation: Yu Tang, Jacopo Niccolo Cerasoni, Emily Yuko Hallett High Resolution "DIY" Photogrammetry - 'HRP' Protocol https://dx.doi.org/10.17504/protocols.io.b53xq8pn

This is an open access protocol distributed under the terms of the **Creative Commons Attribution License** (https://creativecommons.org/licenses/by/4.0/), which permits unrestricted use, distribution, and reproduction in any medium,

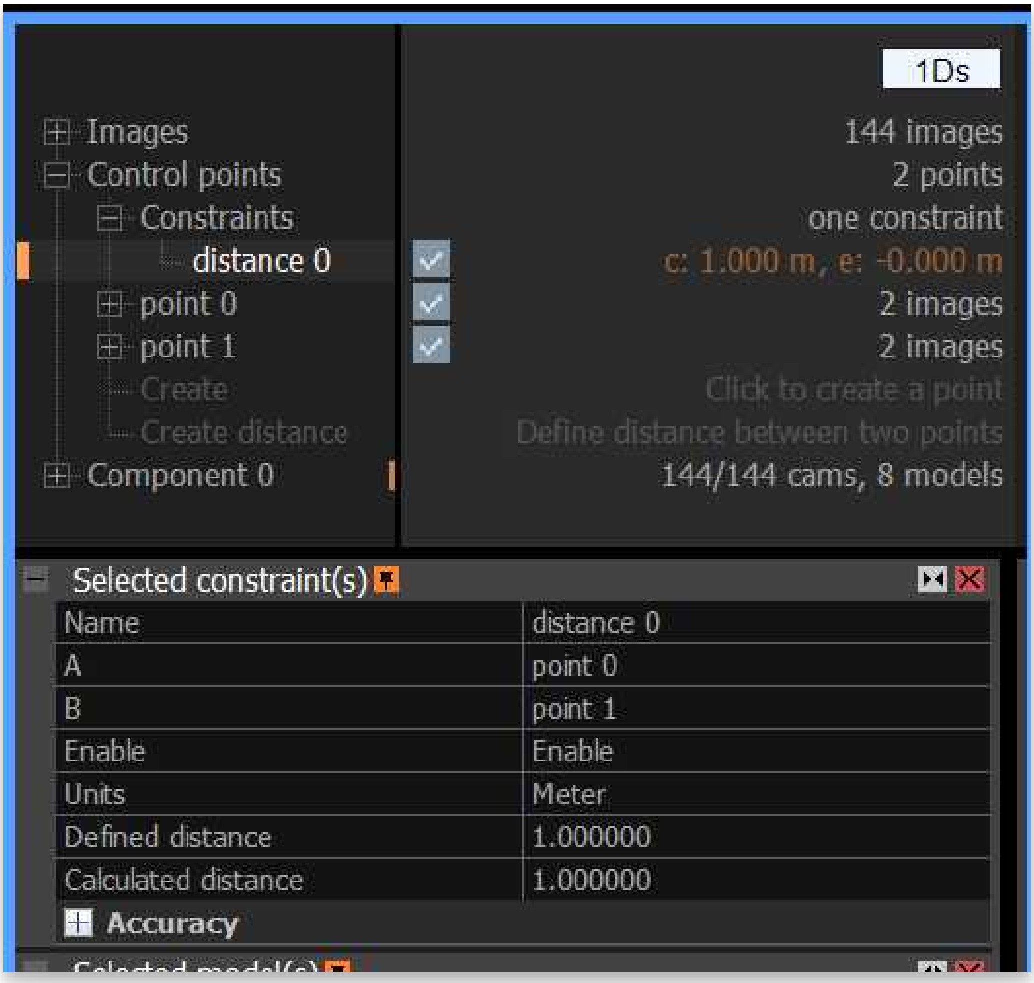

Exporting Model

33 Select the model you want to export in Components.

34 Select "Export - Dense Mesh Model" in the "MESH MODEL TAB".

35 Set a file name and file format and in the next window select "Export Textures" as "Yes".

> If you set 1 unit to 1 cm in distance, the Scale XYZ needs to be set to 1.0.

> ⚠ There are many different types of file formats 3D models can be exported as. The most commons, together with their general use, are:

Citation: Yu Tang, Jacopo Niccolo Cerasoni, Emily Yuko Hallett High Resolution "DIY" Photogrammetry - 'HRP' Protocol https://dx.doi.org/10.17504/protocols.io.b53xq8pn

This is an open access protocol distributed under the terms of the **Creative Commons Attribution License** (https://creativecommons.org/licenses/by/4.0/), which permits unrestricted use, distribution, and reproduction in any medium,

OBJ files, used in this case, is among the most common and widely used. It is a neutral format, meaning it can be used and viewed by multiple softwares. Usefil for exchanging models, use in CAD softwares, and 3D printing application. They can support unlimited colours, and multiple objects can be included in a single file.

STL files is a neutral also an extremely common type of format. Generally used only for 3D printing as this file format only stores geometry and polygon information.

USDZ files are similar to OBJ files, however they are proprietary and can only be created and used within the Apple © ecosystem. They are commonly used for Aritificial Reality (AR) purposes.

36 Click "OK" to finish exporting the model.

Citation: Yu Tang, Jacopo Niccolo Cerasoni, Emily Yuko Hallett High Resolution "DIY" Photogrammetry - 'HRP' Protocol https://dx.doi.org/10.17504/protocols.io.b53xq8pn

This is an open access protocol distributed under the terms of the **Creative Commons Attribution License** (https://creativecommons.org/licenses/by/4.0/), which permits unrestricted use, distribution, and reproduction in any medium,

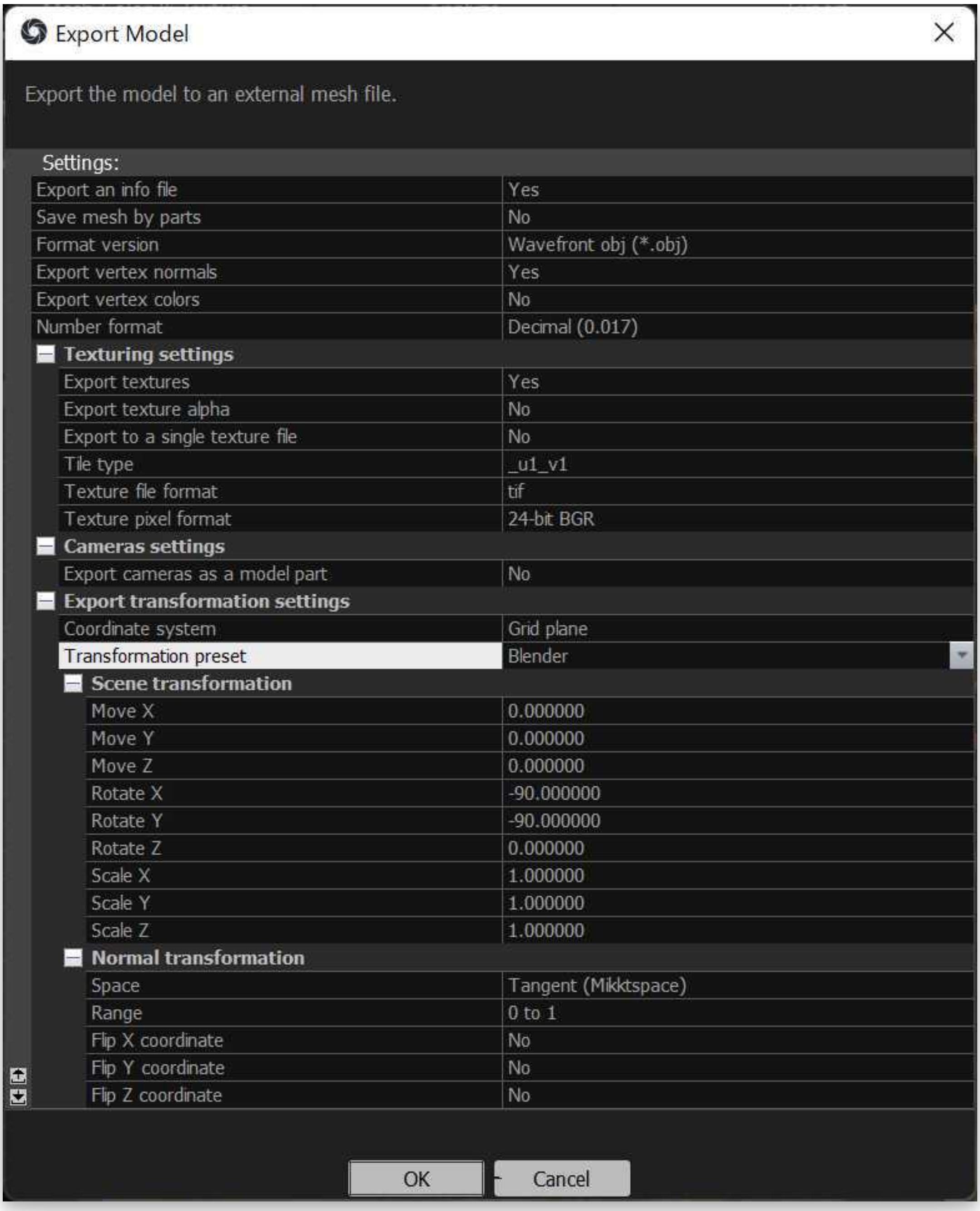

Final Model

Citation: Yu Tang, Jacopo Niccolo Cerasoni, Emily Yuko Hallett High Resolution "DIY" Photogrammetry - 'HRP' Protocol
https://dx.doi.org/10.17504/protocols.io.b53xq8pn

This is an open access protocol distributed under the terms of the **Creative Commons Attribution License** (https://creativecommons.org/licenses/by/4.0/), which permits unrestricted use, distribution, and reproduction in any medium,

37 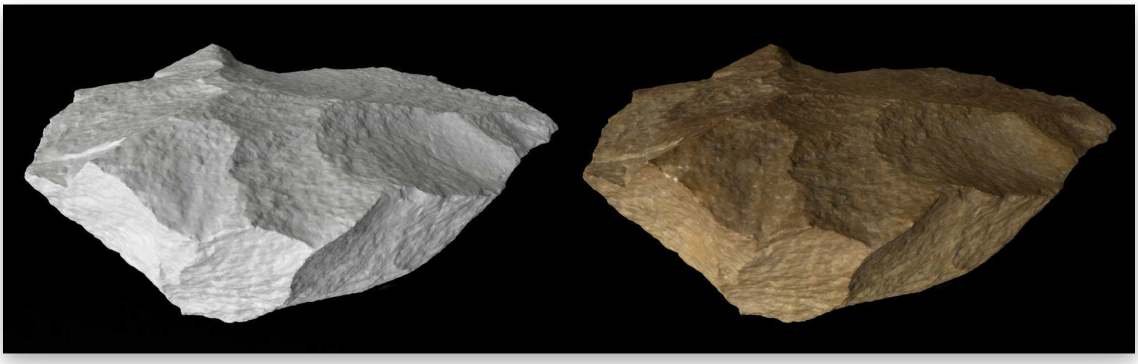

Final model. Left, model with no texture. Right, model with texture.

If you wish to produce and share your own 3D models, we suggest using:

1. **Sketchfab** (https://sketchfab.com) for non-scientific or low polygon-count models. This platform has restricted upload sizes depending on subscription plans. However, a free account with limited monthly number of model uploads can be easily created.

2. **MorphoSource** (https://www.morphosource.org/) for high quality and high polygon-count scientific models. This platform has no upload limit in terms of file size or number. To upload here, however, you will need to apply to become a Morphosource contributor.

38 Final render video **with texture**:

Final render **without texture**:

39 Final 3D model **with texture**:

Citation: Yu Tang, Jacopo Niccolo Cerasoni, Emily Yuko Hallett High Resolution "DIY" Photogrammetry - 'HRP' Protocol https://dx.doi.org/10.17504/protocols.io.b53xq8pn

This is an open access protocol distributed under the terms of the **Creative Commons Attribution License** (https://creativecommons.org/licenses/by/4.0/), which permits unrestricted use, distribution, and reproduction in any medium,

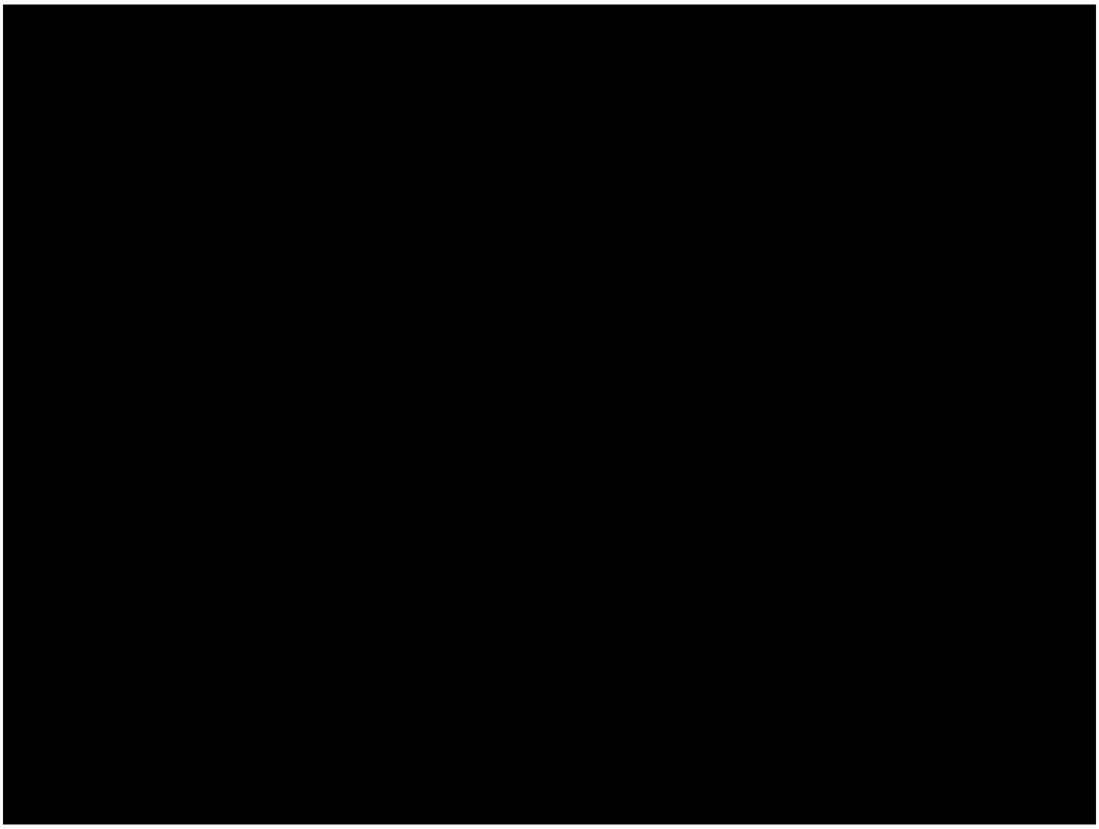

Final 3D model **without texture**:

Citation: Yu Tang, Jacopo Niccolo Cerasoni, Emily Yuko Hallett High Resolution "DIY" Photogrammetry - 'HRP' Protocol https://dx.doi.org/10.17504/protocols.io.b53xq8pn

This is an open access protocol distributed under the terms of the **Creative Commons Attribution License** (https://creativecommons.org/licenses/by/4.0/), which permits unrestricted use, distribution, and reproduction in any medium,

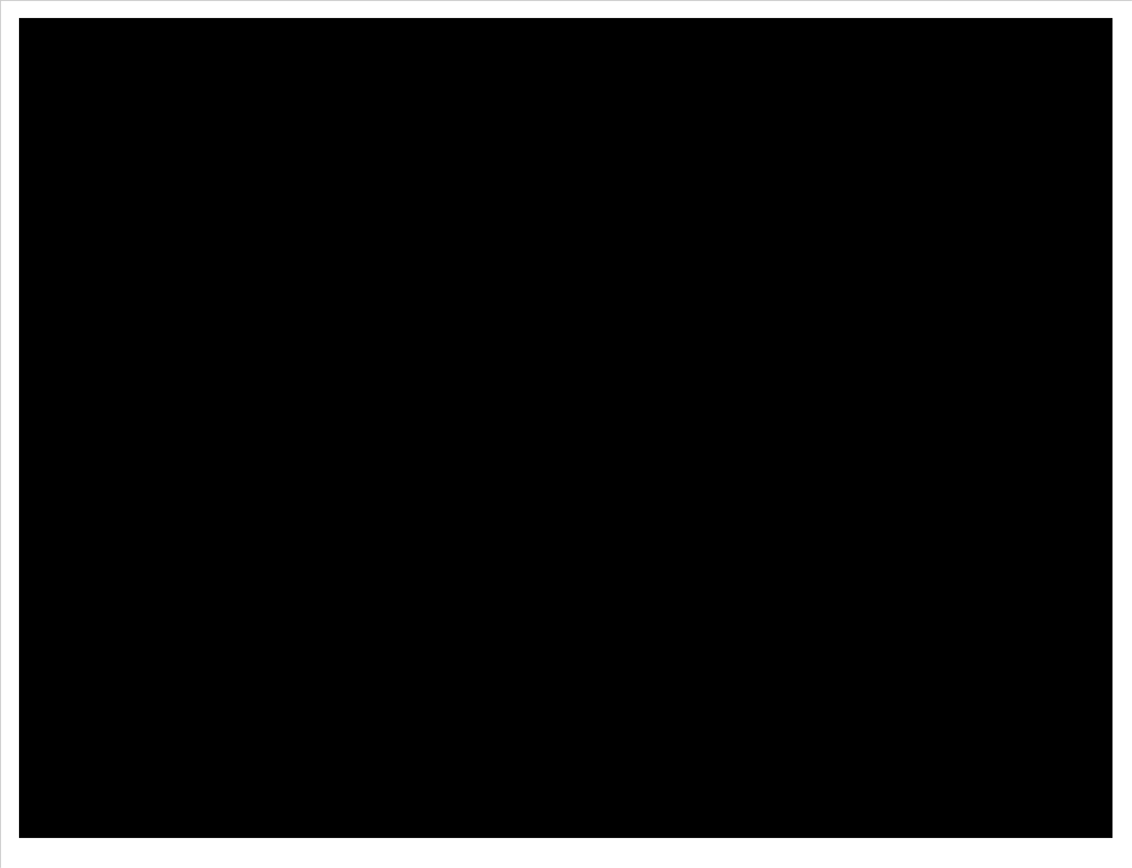

40 Below, you can find the downloadable files of the final model:

Model in obj. format -> Final_Stone_Tool.obj

Model texture in .tiff format -> Final_Texture.tif

Try doing it yourself!

41 **Download the attached .zip file, and try following each step to produce your very own 3D model of this quartzite levallois core!**

Start from *Step 15* if you wish to start by processing the RAW images, or skip to *Step 26* if you want to go straight to the photogrammetric processing!

Teaching Example_Tang, Cerasoni, Hallett, 2022_HRP.zip

(The following .zip file contains the original RAW images used for producing the 3D model presented in this protocol)

Citation: Yu Tang, Jacopo Niccolo Cerasoni, Emily Yuko Hallett High Resolution "DIY" Photogrammetry - 'HRP' Protocol https://dx.doi.org/10.17504/protocols.io.b53xq8pn

This is an open access protocol distributed under the terms of the **Creative Commons Attribution License** (https://creativecommons.org/licenses/by/4.0/), which permits unrestricted use, distribution, and reproduction in any medium,
