# Supplementary material for: Do-It-Yourself digital archaeology: Introduction and practical applications of photography and photogrammetry for the 2D and 3D representation of small objects and artefacts
Source: PLoS One. 2022 Apr 15;17(4):e0267168. doi: 10.1371/journal.pone.0267168 (PMC9012351; doi:10.1371/journal.pone.0267168)
Supplement: Attachment — Submitted filename: Response to Reviewers Guide.docx [file pone.0267168.s004.docx]

**RESPONSE TO REVIEWERS GUIDE**

**Long Title:** Do-It-Yourself Digital Archaeology: Introduction and Practical Applications of Photography and Photogrammetry for the 2D and 3D Representation of Small Objects and Artefacts

**Short Title:** DIY Digital Archaeology: Photography and Photogrammetry of Small Objects and Artefacts

**Authors:** Jacopo Niccolò Cerasoni, Felipe do Nascimento Rodrigues, Yu Tang, Emily Yuko Hallett

Legend:

- General text
- Reviewer and Editors comment
- Author’s comment
- Author’s applied changes

**Reviewer: 1**

The text in my opinion presents very broadly practice guidelines potentially useful for archaeologist just making their first steps into artifact photography or 3d modeling of archaeological artifacts. I do agree with the basic premise of the presented text, yet I do not believe it is in fact a scientific article nor is it a detailed description of a workflow.

Dear Reviewer. We thank you for your time and efforts on reviewing our manuscript. Overall, we do agree that our manuscript is not a scientific article. The manuscript was submitted as a new PLOS One format (lab protocol), and for this reason was not conceived from its very inception as a scientific article in its common form. A lab protocol manuscript is meant to be an accompanying text to the protocols present on protocols.io and included as supplementary information. To this end, we used this manuscript to offer a general background to fully understand and apply the protocols we included. Regarding the lack of a detailed description of a workflow we unfortunately will have to disagree, as we believe the two supplementary protocols offer in detail step-by-step processes for the correct 2D and 3D representation of artefacts.

**Note 1**: We would like to point out a possible misunderstanding regarding the scope of the paper, which seems to be in contrast with the scope Reviewer 1 has of this manuscript. As Reviewer 2 correctly assumed this manuscript is not meant to be a state-of-the-art evaluation of the methods presented, but rather an introductory text for archaeologists and students who do not have extensive experience in the presented fields. By combining tested and clearly explained processes, we hoped to create a set of protocols that could aid in the production of high quality 2D and 3D representations of artefacts by anyone interested.

We believe the following main text section will support our statements:

These methods were developed to allow anyone to easily and inexpensively produce very high-quality images and models for any use, from simple graphic visualisations to complex analytical, statistical and spatial analyses.

While a variety of publications on artefact photography and small object photogrammetry already exist, with the application of the ‘SOAP’ and ‘HRP’ methods it is expected that users will be able to produce high-quality and publishable two- and three-dimensional visualisations of their archaeological artefacts independently and without the necessary dependency of other methodological sources. Furthermore, with enough practice over time and access to the softwares listed above, anyone who is interested in archaeological material culture, whether for personal, educational, or professional reasons, will be able to do so while keeping time and costs as efficient and low as possible.

The paper combines authors' view on both object photography and photogrammetry 3d modeling of artifacts. Both these areas are huge and diverse fields in their own rights. Both can be treated as separate disciplines of art, craft and science and each can be studied full time to the MA/M.Sc. level and beyond on various universities around the world at readily available courses. To attempt to sum up this amount of knowledge into one paper is a difficult task. Possibly bound to present summaries simplified beyond the level which can be supported by data. Thus, the paper presents no data.

We absolutely agree that what we tried to do in this manuscript is indeed a very difficult task. Furthermore, we generally agree that our manuscript presents no data, however we believe that this is not an issue. As presented in Note 1, our manuscript sets to present novel protocols rather than presenting specific research questions or data-driven analyses. In addition, while there are university courses on these subjects, we disagree that these are "readily available” around the world. Our protocols and manuscript were created to remove barriers for archaeologists in areas of the world where such courses are not available.

In my opinion, the text should be rewritten as at least two separate ones where authors could focus separately on each subject.

Thank you for your comment. We agree that the two presented protocols could very well be divided into two separate manuscripts. Nevertheless, a single manuscript presenting both was the best option for us as the first protocol “SOAP”, is a fitting introduction to the second protocol “HRP”.

Firstly comes the premise of Small Object and Artifact Photography -"SOAP" workflow. Most statements which have found its way into the attached protocol should be developed in concise form into the body of the article and the protocol itself should present actual data, preferably a statistical sample.

Unfortunately, it is unclear what the reviewer means by actual data or statistical samples. Nevertheless, the following sentence was included in the Introduction of the manuscript to address any potential confusion towards the lack of inclusion of statistical/dependent analyses in our work:

Quantitative methods of image analysis (see Wang et al., 2004; Bovik, 2005; Simoncelli, 2005) were not applied in our protocols as they fall outside the scope of the applied methods in archaeology that we present here.

References:

Bovik, A., (2005). Handbook of Image and Video Processing (Second Edition), Second Edi. ed, Handbook of Image and Video Processing (Second Edition), Communications, Networking and Multimedia. Academic Press, Burlington.

Simoncelli, E.P. (2005). 4.7 - Statistical Modeling of Photographic Images. In Handbook of Image and Video Processing (Second Edition), A.L. Bovik, ed. (Academic Press), pp. 431-441.

Wang, Z., & Bovik, A. C. (2002). A universal image quality index. IEEE signal processing letters, 9(3), 81-84.

The protocol should also explain actual gains of at least one step of the process which authors believe they have perfected beyond what is available now in practice of professional photographers, in on-line sources or in literature. Possibly focusing on one aspect specifically would also help the authors to explain what in their opinion is the decisive quality of a “good” or “bad” photograph.

Thank you for your comment. Our protocols did not perfect the field of artefact photography beyond already existing professional methods. All of the material present in our SOAP protocol can be found in on-line source, literature, or with professional photographers. What we did, however, was put together a protocol that would make it much easier for interested people to learn this method without having to do in-depth research, therefore easing the learning curve and process. In regards to the differentiation between “good” or “bad” photograph, including our opinions of such a matter would mean including far too subjective perspectives into a manuscript that we tried to keep as objective as possible.

The issues of focus stacking, illumination control, diffraction, lens selection, background choice or background removal etc., each could warrant a discussion and possibly improvement. In its current form, the paper in this part and attached protocol do not convince that following presented advice to the letter will result in any specific improvement. Most statements are generally “common knowledge” for a working photographer and are only new to an archaeologist venturing first into serious photography.

That is exactly the scope of our manuscript. We do not expect working photographers to learn new skills from our protocol. Rather, we hope to offer hints to the professionals who have never worked on scientific photography of artefacts, but most importantly teach beginners all the necessary fundamentals for producing high-quality, publishable, photographs.

This part of work in essence will benefit from restructuring. It is vital to show which part of photographic documentation process can be improved. Also, it is important to introduce a quantitative method to show the actual improvement and dataset (large, statistically significant set of photographs) pointing the areas of improvement.

Thank you for your comment. We do agree that the photographic documentation process can definitely be improved. The section below was already present within the manuscript, which we believe discusses the limitations of the method and potential improvements in the main text (present below). Regarding the inclusion of a quantitative method we unfortunately disagree as it would be far outside the current scope of the manuscript. Nevertheless, we have added references to quantitative methods of photographic analysis (see previous comment for section and references).

The ‘SOAP’ and ‘HRP’ protocols offer clear step-by-step processes that anyone can learn and put into practice. However, it is important to note that a good photograph or a good three-dimensional model will always be just a visual representation of the visualised artefact. For this reason, it is important to note that a good understanding of the artefact’s morphology, technological characteristics and context will always be necessary for the correct interpretation of the visualised material culture. Furthermore, both methods will inevitably encounter limitations depending on the used equipment, workflow variations, and subjective evaluations during their application. Both in terms of photographic and photogrammetric documentation, minor differences will occur depending on a range of variables which will be person- and case-specific. Potentially, improving equipment capabilities, both in terms of hardware and software functionality will automatically result in better and more efficient final products. Increased time spent on practicing the presented methods will also exponentially improve their application and outcomes.

Secondly, part of the text referring to photogrammetry ”HRP” protocol also presents no actual data. Authors did not explain sufficiently in paper nor in protocol what are the markers to measure the quality of a photogrammetric 3d model and how using their specific workflow will make a significant or even measurable difference over just following other workflows, including those proposed by 3d photogrammetry software manufacturers according to this measured parameter. This parameter has to be explained in order for the research to ever be replicated. The improvement of the parameter should also be presented on a statistical sample of processed 3d models. To create such a body of data would be time-consuming, but would give the paper and proposed protocol some credibility.

Thank you for you comments. Once again, we would like to reiterate that the presented paper and supplementary protocols are meant to be a presentation of workflows for people new to the field of 2D and 3D imaging. While there are many photogrammetry software options available, there are cost prohibitive for many archaeologists, and especially those without access to funding. We also seem to not understand what the reviewer means by “parameter”. In terms of the credibility of the protocol, we have included extra clarifying steps (see reviewer 2 comments) and the original RAW images used in the practical example. We hope this will further support the reproducibility of our methods.

As it is now the paper in this part doesn’t present any information and the attached “HRP” protocol is an instructional material no better or worse than any other similar workflow and its usability will only be subjective and dependent on the experience of those who might have used it for their first work in 3d photogrammetry.

We agree on the nature of the presented protocols as instructional materials, and we think this sort of product is extremely needed, especially in terms of detailed workflow such as the one we present. Of course, such methods will always be dependent on subjective aspects characteristic of every person who will try learning and/or applying said methods. However, by providing a workflow that is as detailed and clear as possible, we tried minimising subjective interpretation of the workflows.

**Reviewer: 2**

**Dr. Samantha T. Porter**

More public, citable technical guides in archaeology are desperately needed, especially for newer techniques like photogrammetry, and I thank and commend the authors for their contribution. From a high level, the article does a good job of setting the stage for the two associated protocols. These protocols are well thought out, have many good illustrations, and include lots of important details, but could be reorganized and edited to add more context and be easier to follow for readers who may not be as familiar with photography or 3D scanning. Due to the fact that there are currently not many formal courses on these subjects as mentioned by the authors, it is possible that a significant number of readers will fall into this category. If it has not been done already, having inexperienced users ‘play test’ the protocols could reveal additional ways the protocols could be made more broadly helpful.

Dear Dr. Porter, we thank you wholeheartedly for your comments and reviews. We appreciate very much your thoughts on our manuscript, and its utility in the field of archaeology. Below you will find our responses to your reviews, which we believe make our manuscript and related protocols much stronger and understandable. Also, we would like to note that two of the listed authors have had limited experience with the application of photography and photogrammetry, and have been actively participating in the production of the manuscript so to assure a user-friendly nature of the attached protocols.

Specific comments on the different submitted elements follow.

----

Article Text:

In the abstract, it may be best to specify that photogrammetry refers to 3D reconstruction / “structure from motion” as technically “photogrammetry” could more broadly refer to the general concept of taking measurements from photographs.

Thank you very much for the clarification. We absolutely agree on the need to make the abstract clearer on this point, and have therefore modified it as follows:

Here, we present two new methods, the ‘Small Object and Artefact Photography’ (‘SOAP’) and the ‘High Resolution “DIY” Photogrammetry’ (‘HRP’) protocols. The ‘SOAP’ protocol involves the photographic application of modern digital techniques for the representation of any small object. The ‘HRP’ protocol involves the photographic capturing, digital reconstruction and three-dimensional representation of small objects.

What the authors mean by ‘illustrative’ versus ‘object’ representations of objects in the fourth paragraph of the second page is not exactly clear and could be better defined. What in the authors’ view are the goals of each type of representation?

Thank you for the request of clarification. We understand that our original definitions and descriptions were unclear. We have added a section within the highlighted paragraph to clarify the reviewer’s concern.

A major improvement in the visual representation of archaeological artefacts has been the shift from an illustrative and artistic photographic style, to a more analytical and objective format. While artistic photographic styles are still employed in cases where public outreach and science communication are of primary interest, analytical and objective photography has become essential for the proper representation of archaeological materials and contexts following proper scientific methods. Examples of this are the increased use of satellite imagery as a primary data source for archaeological surveying [7] and high-resolution microscopic photography as an essential method for the identification and recording of past tool uses [8].

In the third paragraph on page 4, I would add LiDAR (perhaps both terrestrial and aerial) to the list of 3D recording methods. It may be more appropriate to use e.g. as opposed to i.e. This is also true perhaps of the list of types of material culture in the second paragraph of the introduction.

Thank you for your comment. The suggested edits have been added to the manuscript.

As many of the software applications described in the protocols are not free (Adobe Photoshop, Reality Capture, and ArcGIS for example) it is maybe not appropriate to highlight free software in the expected results section. The authors could state that much of the software is likely already accessible to folks in an academic environment, or that their photography protocols could be used with free alternatives to the software applications described. It’s also quite minor, but in my experience practice over time does definitely improve results for both artifact photography and photogrammetry.

Thank you very much for your comments. We agree that highlighting free softwares is definitely a good addition to the manuscript. A selection of free alternative softwares has been added to the end of the “Introduction”. Below is the added section:

Although most of the used softwares in the ‘SOAP’ and ‘HRP’ protocols are readily available in academic environments, these methods can be applied to any other non-subscription based softwares with similar features. In this regard, free and/or open-access softwares can be readily used, albeit with minor changes in the application of some of the presented steps depending on the used software’s functionalities. For raster-based softwares used for both photography and photogrammetry, as Adobe Photoshop ©, free to access softwares such as GIMP © and Krita © or single-purchase products such as Affinity Photo can be used. For 3D Reconstruction photogrammetric softwares, such as RealityCapture ©, a good free and open-source alternative is Meshroom ©.

We have also included, within “Expected results”, the importance of practice for improving results of artifact photography and photogrammetry.

Furthermore, with enough practice over time and access to the softwares listed above, anyone who is interested in archaeological material culture, whether for personal, educational, or professional reasons, will be able to do so while keeping time and costs as efficient and low as possible.

-----

‘SOAP’ Protocol:

It may be worth mentioning copy stands as a mounting option, as in my experience many academic departments have these laying around and they can be more stable than many models of tripod.

Step 2 now includes copy stand as a mounting option.

For readers without much experience, it may be worth defining a macro lens and stating why it is most suited to this type of photography. They could also refer readers to section 8 (Step 2).

Thank you for this comment, a definition of lenses would be absolutely useful. We have included an introductory step defining and describing lenses and lens selection (Step 1.1):

Arguably the most important part of a camera is the lens. Before continuing with this method, we suggest the reader understand what type of lenses exist and which one works best for its use. In this case, we suggest using a macro lens. Find below definitions and descriptions of Macro Lens and commonly used standard zoom lens:

**Macro Lens:** A Macro Lens is a type of lens that allows very close up shots of the subject. In many cases, it requires close proximity to the subject being photographed but in return, it offers high levels of detail, which are difficult to be acquired with regular lens. This in turn, makes macro lens ideally suited for small object and artefacts photography.

**Standard Zoom Lens:** Standard Zoom Lens is a highly variable type of lens, allowing for the capture of landscapes, people, buildings and small objects and artefacts. Although it may not perform as well as a macro lens, its flexibility makes it an ideal lens for many archaeology professionals, projects and institutions, hence, one of the most commonly available lens types.

Ultimately, the choice of a lens should be reflected against the questions and needs of the archaeologist/photographer. If highly detailed close-up photographs are required, a Macro Lens is recommended. However, if illustrative photographs are required, a Standard Zoom Lens is more than capable to achieve good and usable photographs.

The authors could also suggest that readers use a two or ten second timer on their camera if remote control is not possible. This can minimize blurriness due to shake. Tethering to a computer is another option. The reasons why this should be done should be stated upfront (Step 2.2).

A note in Step 2 was added to account for remote control timer:

In case remote control is not available, the use of the camera timer (2-10s) is recommended as an option to avoid blurriness from shaking when operating it on the tripod/mount.

Satin paper and velvet are mentioned, but it is not explained why these materials are good options. It would be good to add an explanation (e.g. they are not reflective) (Step 3.1).

Step 3 has been modified as follows:

Place background material. Satin paper or velvet are preferred given their non-reflective properties.

There is a missing word in the explanation of the RAW format (...preferred as they are the least processing…” The description of RAW and TIFF files as being more ‘archival’ could be helpful. Inexperienced users may not understand what compression is and how it can degrade an image. I would suggest adding an explanation. (Step 6)

The definition of RAW image formats has been rewritten:

RAW image formats (CR2, CRW, NEF, PEF...) are always preferred as they undergo the least amount of digital processing, with little to no loss of visual details.

A note has also been added defining compression:

In photography, compression is the process of minimizing the digital size of a graphical element (photograph) without degrading the quality of an image to an unacceptable level. Due to this, both RAW and TIF formats are very good for archival purposes.

I would move a discussion of the “exposure triangle” earlier in section 8 to give context to the suggested camera settings (aperture, shutter speed, and ISO). Some sort of visualization of this concept would be helpful.

An explanation and graphical representation of the exposure triangle has been added in Step 9:

Exposure is the amount of light which reaches the camera sensor. This is dependent on the relationship between the three main functions discussed previously, which include:

1. Aperture

2. Shutter Speed (or Time Value)

3. ISO

Adjusting these three separate functions will result in a certain amount of light reaching the sensor. Furthermore, exposure can be further altered using the in-camera exposure function.

Note: Although it is possible to set a determined exposure value either as positive (lighter) or negative (darker) values, the exposure setting should always be set at 0 when possible. This is because further digital processing will be applied when exposure is directly set onto the camera, decreasing image quality. Exposure should therefore be adjusted only by regulating aperture, shutter speed and ISO.

Figure: 'Exposure Triangle' - Graphical representation of the relationship between the three main variables influencing image capture and described in Step 9.

I think it would make sense to discuss ISO before the shutter speed, as it should usually be set at a low value, while exposure time is more likely to be variable depending on things like material properties, ambient light in the environment, etc.

Thank you for your comment. ISO has been moved into Step 11, with shutter speed becoming Step 12.

This is not going to affect most users, but some lenses do not have both a focus and zoom ring and focus must be adjusted by moving closer / further to the subject. For example our lab uses a Laowa 25mm f/2.8 2.5-5X Ultra-Macro Lens for very up close photography / photography of very small objects. Going into this in detail is likely not worth it, but it may be helpful to mention that the example depicted applies to most but not all lenses (Step 10).

We agree that although it wouldn’t be of interest to everyone, an inclusion of this possibility is appropriate, and we have therefore included the following note in Step 13:

Note that some lenses do not have both focus and zoom ring. In these cases, focus is adjusted by moving the camera closer/further to the object. The example illustrated above applies to most, but not all lenses.

A visual of what ‘exposure’ means on camera and what it does would be helpful, as shutter speed is often colloquially referred to as exposure (Step 12)

We believe this comment has been resolved by the inclusion of an exposure triangle and further explanations in Step 9.

An explanation of white balance would be helpful during the photography instructions (and not just the editing instructions), especially if people do not end up shooting RAW.

A step specific to white balance with detailed explanation has been included, and can be seen in Step 8:

White Balance

White balance is the adjustment of the colour temperatures captured by the lenses. On the camera, this is a process that can be adjusted in three ways:

1. Automatic: Auto adjusted during capture

2. Pre-set: Predetermined categories built in the camera (e.g. 'Cloudy', 'Incandescent', 'Direct Sunlight')

3. Manual: Some cameras come with the option to manually choose a colour temperature (Values in Kelvin)

These on-camera options can be useful if a quicker acquisition and processing is required.

Lastly, White Balance can be also changed on the Post Processing phase by: manually adjusting the values on post processing software (see step 18).

Some consider non-linear adjustments like curves and levels to be non-ideal for scientific publishing as they affect certain pixels more than others. See for example Rossner and Yamada 2004 (https://doi.org/10.1083/jcb.200406019). Photo filters are a linear alternative, but must be done by eye. An alternative would be to have users disclose any manipulation done on the image in publication (Step 18).

Sincere thanks for your comment. We were not aware of the cited study and appreciate it very much. We have included the citation, together with an explanatory text in a note included in Step 18:

Please note that the following method describes a non-linear colour adjustment of pixels within a photograph. Studies have shown that this method can at times be non-ideal for scientific purposes (Rossner and Yamada 2004; https://doi.org/10.1083/jcb.200406019). Alternatively, photo filters can be used to achieve a linear adjustment, although they require subjective judgement.

If the method described here is used for scientific publication, we suggest disclosing that this manipulation technique was used.

It could be helpful to suggest that readers also save their work as a PSD file if it needs additional manipulation later on (for example to be able to copy the cut out object and use it in a collage later on) (Step 27).

Thank you for this very useful comment. We have included it as a note on the final step (28):

.TIFF, .JPG, .PNG are useful formats once the image is finished. However, it is recommended to have a version saved on the native format of the image processing software used (i.e. .PSD on photoshop, .XCF on GIMP or equivalent) as that allows further processing of the image if required.

-----

‘HRP’ Protocol:

A list of software in addition to hardware at the start of the protocol would make the article easier to read.

**Hardware**

  DSLR or mirrorless Camera

  Tripods

  LED light or flash

  Flash Diffuser

  TurnTable

Scale

  White or black background cloth

  Colorchecker (if colour correction is required)

  Remote controller (if not using the mobile phone software remote control)

**Software** (generally paid softwares are faster and more efficient)

Photographic Processing Software

  Paid software: DxO Photolab/Adobe Photoshop/Adobe Lightroom/CaptureOne

  Free software: Darktable/Rawtherapy/Libraw/DCRaw

Photogrammetry Software

  Paid software: Realitycapture/Agisoft Metashape

Free software: Meshroom

That you for the observation, a list has been added under the “Materials” section together with the hardware.

It would be helpful if the authors explained why using a cell phone is not as accurate and precise (e.g. lack of manual control) (End of Step 1).

Thank you for the request of clarification. We have modified the section at the end of Step 1 to fully explain the limitations of mobile phone use:

If using a mobile phone, scans will never be as accurate and precise and they could be when using a digital camera.

Although it is possible to take excellent quality scans with a mobile phone, several considerations and extra steps are necessary to achieve this. As mobile phones to not usually have manual control for photography, extra-party softwares are required. Furthermore, photographs should be taken in RAW (DNG) format, using a remote control and adequate lighting.

Furthermore. due to the small sensor size of mobile phone cameras, it is often necessary to take more pictures or to get closer to the object to achieve high-detail models. Although this is not problematic when wanting to apply this method to simple objects, complex or more detail objects will be extremely difficult to correctly photograph. In turn, DSLR or mirrorless camera will give better results with less time and effort.

It would be helpful to explain why a solid color background is ideal (e.g. it helps separate the target object from the background). I am not familiar with Reality Capture, but for Agisoft Metashape for example it allows for easier masking. It also may be helpful to describe the importance of contrast between the object and background as was done in the ‘SOAP’ protocol (Step 2).

Thank you for your comment. To address your proposed change we have divided Step 2 into two sub-steps, and included a detailed note discussing background color:

A solid color for the background will aid the subsequent processes in a variety of ways. They are:

1. Help separating the photographed object from the background.

2. The photogrammetric software will hardly detect any feature or key point from the background, therefore making the automatic matching process easier.

3. A solid color will be easier to mask on the photogrammetric software.

Furthermore, contrast between the object and the background, set by adjusting the lighting, will aid in the isolation of the photographed object from the background. For further explanations on the process of background, lighting and contrast selection see Step 3 of the 'SOAP' protocol (dx.doi.org/10.17504/protocols.io.b53zq8p6)

As mentioned in my comments on the ‘SOAP’ protocol, in order to reduce blurriness from motion, setting the camera on a two second timer is an alternative if a remote shutter is not a possibility (Step 3).

The note on Step 3 has been modified as follows to include the use of a timer:

It is recommended to use the remote control shutter with between 2 to 10 second timer to avoid shake and bluriness. If the camera does not include one, remote control can be accessed via WiFi or Bluetooth; both Android and iOS universal applications exist for this purpose.

Photography terms such as aperture are not as well defined in this protocol as they are in the other. Perhaps the reference to ‘SOAP’ could be moved earlier.

We agree that the protocol lacks of detailed definitions and explanations. To counter this, we created a new Step (5, present below) which references the SOAP protocol:

The following steps (6-14) present photographic methods and theories which are fundamental for the correct capturing of photographs for photogrammetric purposes. Further detailed explanation for all the discussed techniques and functions can by consulting the "Small Object and Artefacts Photography" protocol (dx.doi.org/10.17504/protocols.io.b53zq8p6).

It is my understanding that for many lenses diffraction begins at f/11, and that thus f/16 should be avoided. See for example Verhoeven 2016 (Basics of Photography for Cultural Heritage Imaging, <https://biblio.ugent.be/publication/8050621/file/8050622.pdf>).

Thank you very much for your comment and the reference. We do agree that an F/11 aperture is more commonly used and should in fact be the suggested aperture for the protocol. Nevertheless, we believe that in certain circumstances F/16 is still a valid option, albeit with a high-quality lens. We therefore changed Step 8 to address this:

Regular Photogrammetry usually selects an aperture between F/5.6 and F/16. Different aperture sizes depend on the focal length and the size of the subject. Too small aperture will cause diffraction and reduce the quality of the photo, while too big aperture will reduce the depth of field.

Generally, for small objects an aperture of F/11 is suggested. Higher apertures such as F/16 are necessary in some cases if the object is too small, however this requires a high-quality lens (e.g. SEL90M28G) so to avoid diffraction.

An explanation that high ISO can result in noise being added to an image may be helpful here. A sample image would be particularly welcome.

That you for the very welcome comment. In Step 9 we have added a short explanation of high ISO causing noise, and have also added a set of sample images with different shutter and ISO values.

There is a typo where Rawdigger is spelled Rawfigger. (Step 8).

Thank you for noticing. The typo has been corrected.

There is a typo at the start of Step 11 (I believe “repeat rolling” should be “repeat rotating”). A distinction perhaps between rotating the turntable and turning the object upside down should be made, as it is currently confusing. Diagrams of the different photography options (i.e. turning the object around vs moving the camera) would be very helpful.

Thank you for noticing the mistake, “rolling” has been changed to “rotating”. Several changes and reorganization of the steps have been made, please see new steps 11-12.2. Also, a diagram has been created, hoping to make the discussed methods easier to understand:

Visual representation of Method 1 and 2 explained in *Step 12*. In grey the rotating turntable is shown. In red are the virtual positions of the camera lens, showing the variation in height at constant distance for each of the two methods. Note that Method 1 is used for an object than can be placed comfortably on different faces, while Method 2 is used for an object which can be placed without the aid of external holders only on two separate faces.

For scientific purposes, in my opinion a scale should be necessary and not just recommended (Step 11.3).

Thank you for your comment, and we agree that it should always be included. The note in Step 13 has been updated as follows:

It is essential to include a scale in the photographs of the selected object. Ideally, the scale should be fully visible in at least 4 or 5 of the photos so to aid in the alignment of all the photographs.
I would suggest adding that objects should be rotated 20 degrees between images as expressing this as 18 times per rotation isn’t necessarily intuitive (Step 12).

Is there a reason DxO PhotoLab is used for RAW imaging processing in this workflow, while Adobe Photoshop is used in the other workflow? I do like the suggestion of LibRaw as a free alternative, and wonder if it is worth mentioning that in the SOAP protocol? In both cases, if readers do not have access to software to edit RAW files it may be worth stating that they can still achieve good results (Step 14).

Thank you for the very fair question. The SOAP protocol uses Adobe Photoshop as the rest of the processing steps are carried out on the same software, therefore offering a more streamline workflow without using too many software. This is not the case with the HRP protocol, therefore suggesting the RAW processing software (DxO PhotoLab) which we believe is the best option for the specific workflow.

In regards to the second part of the comment, we agree and have included a note in Step 15:

Please note that the processing of RAW images is optional. Although the following steps (15-25) will result in the best possible outcome, the use of unprocessed RAW images will still produce a good final result.

The adjustments in steps 16 and 17 seem like they could be useful for photogrammetric processes, but are non-linear and thus not ideal for scientific use (see again Rossner and Yamada 2004 - https://doi.org/10.1083/jcb.200406019). It may be worth being explicit about the need to disclose this workflow when publishing models created using this protocol.

Thank you for raising this issue. We have included a note (as in the SOAP protocol) in step 18 stating the following:

Please note that the following method describes a non-linear colour adjustment of pixels within a photograph. Studies have shown that this method can at times be non-ideal for scientific purposes (Rossner and Yamada 2004; https://doi.org/10.1083/jcb.200406019). Alternatively, photo filters can be used to achieve a linear adjustment, although they require subjective judgement.

If the method described here is used for scientific publication, we suggest disclosing that this manipulation technique was used.

Since ‘Metashape’ is a newer name for the software, I would refer to it by its full name of ‘Agisoft Metashape.’ Including some advantages and disadvantages of the applications mentioned would be welcome (Step 14).

Thank you for noticing our mistake. We have updated the name of the software to “Agisoft Metashape”. Furthermore, a note has been added giving more detail on the different pros and cons of the mentioned softwares:

Other softwares such as Agisoft Metashape or 3DF Zephyr can also be used. They are both extremely efficient and usable softwares, with lower requirements of CPU memory, and being able to create 3D meshes with much lower CPU power. However, in both cases a higher degree of post-processing will be required as they tend to produce higher noise during the production of 3D models compared to RealityCapture.

The protocol should define ‘too many faces’ for inexperienced users. This could include an explanation that a high number of polygons can be difficult to load and manipulate on standard computers for example (Step 29). A suggestion for an upper limit on the number of polygons could help readers.

Thank you for requesting this clarification. We understand that as it was the step was unclear. We have therefore added the following note clarifying the requested points:

Faces are simply the individual polygons which, when put together, form the surface of the 3D model.

Generally, for online viewing of 3D models a maximum of 20,000 t0 30,000 faces, or polygons, should be considered.

In this case, given the scientific and measurement purposes of the developed model, several millions of faces can be reached.

New users may not know what a texture map is, so an explanation and even an image could be added to make this clearer (Step 30.1).

Thank you for the very fair comment. We added a note with an explanation of texture map and an explanatory image:

3D Texturing is the creation of a 2D image (texture map) by wrapping it a around a 3D object and defining how light would affect it.

Texture maps can be projected onto a 3D object allowing to look at a previously blank model with a texture, or projected image onto it.

An explanation of different 3D file formats would be welcome in the export section, as was done for 2D images in the ‘SOAP’ protocol.

We appreciate the comment and have added the follow note in Step 35:

There are many different types of file formats 3D models can be exported as. The most commons, together with their general use, are:

**OBJ (.obj)**, used in this case, is among the most common and widely used. It is a neutral format, meaning it can be used and viewed by multiple softwares. Usefil for exchanging models, use in CAD softwares, and 3D printing application. They can support unlimited colours, and multiple objects can be included in a single file.

STL files is a neutral also an extremely common type of format. Generally used only for 3D printing as this file format only stores geometry and polygon information.

USDZ files are similar to OBJ files, however they are proprietary and can only be created and used within the Apple © ecosystem. They are commonly used for Aritifìcial Reality (AR) purposes.

The inclusion of the video renders and interactive 3D models is great! A discussion of different options for sharing 3D models would be welcome for newer users, but I think not absolutely necessary to this particular protocol.

Thank you very much! As suggested we added a short note on our two favourite model sharing platform, please find it below:

If you wish to produce and share your own 3D models, we suggest using:

1. **Sketchfab** (https://sketchfab.com) for non-scientific or low polygon-count models. This platform has restricted upload sizes depending on subscription plans. However, a free account with limited monthly number of model uploads can be easily created.

2. **MorphoSource** (https://www.morphosource.org/) for high quality and high polygon-count scientific models. This platform has no upload limit in terms of file size or number. To upload here, however, you will need to apply to become a Morphosource contributor.

For scientific purposes, users will need to record metadata about their photography and processing steps (e.g. number of photos used, software settings, etc.). This should be mentioned somewhere in the protocol.

Thank you for the comment. We absolutely agree and have added a mention of it in the Guidelines and Warnings Section:

If you wish to use this method for scientific purposes, please remember to **record your metadata and workflow**. Make sure to keep notes on (1) number of photographs taken, (2) camera settings, (3) software functions/settings, and any other information which will be useful at a later date.

This will allow you to be able to produce and write documents and methodological texts at the highest possible degree of precision, making sure to not forget or ignore any useful piece of information.

Finally, if possible I would encourage the authors to include a sample dataset of RAW or pre-processed images so users can experiment with the Reality Capture portion of the protocol without having to capture their own images. This would be particularly useful for example as a teaching resource for courses on digital archaeology.

Thank you very much for the amazing idea. We have included a final step, titled “Try doing it yourself!”, where we included the text below, and attached a .zip file containing all the original RAW images used in the practical example of the protocol.
